# *Pantoea* Bacteriophage vB_PagS_AAS23: A Singleton of the Genus *Sauletekiovirus*

**DOI:** 10.3390/microorganisms9030668

**Published:** 2021-03-23

**Authors:** Emilija Žukauskienė, Monika Šimoliūnienė, Lidija Truncaitė, Martynas Skapas, Algirdas Kaupinis, Mindaugas Valius, Rolandas Meškys, Eugenijus Šimoliūnas

**Affiliations:** 1 Life Sciences Centre, Department of Molecular Microbiology and Biotechnology, Institute of Biochemistry, Vilnius University, Saulėtekio av. 7, LT-10257 Vilnius, Lithuania; emilija.zukauskiene@bchi.stud.vu.lt (E.Ž.); monika.simoliuniene@gmc.vu.lt (M.Š.); rolandas.meskys@bchi.vu.lt (R.M.); 2 Center for Physical Sciences and Technology, Saulėtekio av. 3, LT-10257 Vilnius, Lithuania; martynas.skapas@ftmc.lt; 3 Proteomics Centre, Institute of Biochemistry, Life Sciences Centre, Vilnius University, Saulėtekio av. 7, LT-10257 Vilnius, Lithuania; algirdas.kaupinis@gf.vu.lt (A.K.); mindaugas.valius@bchi.vu.lt (M.V.)

**Keywords:** *Pantoea agglomerans*, vB_PagS_AAS23, siphovirus, cold-adapted bacteriophages, *Sauletekiovirus*, *Drexlerviridae*

## Abstract

A cold-adapted siphovirus, vB_PagS_AAS23 (AAS23) was isolated in Lithuania using the *Pantoea agglomerans* strain AUR for the phage propagation. The double-stranded DNA genome of AAS23 (51,170 bp) contains 92 probable protein encoding genes, and no genes for tRNA. A comparative sequence analysis revealed that 25 of all AAS23 open reading frames (ORFs) code for unique proteins that have no reliable identity to database entries. Based on the phylogenetic analysis, AAS23 has no close relationship to other viruses publicly available to date and represents a single species of the genus *Sauletekiovirus* within the family *Drexlerviridae*. The phage is able to form plaques in bacterial lawns even at 4 °C and demonstrates a depolymerase activity. Thus, the data presented in this study not only provides the information on *Pantoea*-infecting bacteriophages, but also offers novel insights into the diversity of cold-adapted viruses and their potential to be used as biocontrol agents.

## 1. Introduction

*Pantoea* is a genus of rod-shaped Gram-negative, nonsporulating, yellow-pigmented and highly diverse bacteria of the family *Enterobacteriaceae*. Although members of this genus have been found to predominate in the phyllosphere of various plants, both as epiphytes and endophytes, *Pantoea* have been isolated from many aquatic and terrestrial environments, as well as in association with insects, animals and humans [1,2,3]. Some strains of *P. agglomerans* have been used for a variety of biotechnological applications including biocontrol against phytopathogenic bacteria and fungi, bioremediation, and therapeutic products [4,5,6]. On the other hand, *Pantoea* isolates that are opportunistic pathogens of humans, animals and plants have been reported as well [7]. Despite the importance of bacteriophages in shaping the general biology, ecology, and evolution of bacteria, and their promising use as biocontrol agents against pathogenic bacteria [8,9,10], *Pantoea*-infecting viruses remain underexplored to date.

Only 14 bacteriophages with completely sequenced genomes, annotated as *Pantoea* phages, have been published or deposited in Genbank to date (Table S1) [11,12,13,14,15]. Nevertheless, it has been shown that a number of *Erwinia* bacteriophages are active on bacteria from the genus *Pantoea* [16,17,18,19,20,21,22,23]. It is not surprising given that *Pantoea* and *Erwinia* form a monophyletic group within the family *Erwiniaceae* [1,24], and some broad-host range bacteriophages are able to infect phylogenetically closely related bacterial species [25]. For example, a broad-host-range lytic siphovirus, phiOT8, which has been isolated on *Serratia marcescens*, infects *Pantoea agglomerans* productively [26].

In this study, we present biological characteristics and a complete genome analysis of *Pantoea agglomerans*-infecting bacteriophage vB_PagS_AAS23 (below referred by its shorter name AAS23). AAS23 shows a low-temperature plating profile, with an optimum temperature for plating of about 22 °C, and an ability to form plaques even at 4 °C. Phylogenetic analysis indicates that phage has no close phylogenetic relationship with other bacteriophages and is a single member of the genus *Sauletekiovirus* within the family *Drexlerviridae*. Notably, bioinformatics analysis reveals that AAS23 is a lytic virus, as none of the predicted gene products of phage shows sequence homology with integration-related proteins. Additionally, none of the antibiotic resistance determinants, or virulence factors like exotoxins, which can be spread through the horizontal gene transfer, and can convert harmless bacteria into dangerous pathogens, have been detected in the genome of AAS23. Thus, the data presented here not only extend our knowledge on morphology, physiology and genetic diversity of *Pantoea* phages but also imply that AAS23 potentially could be used as phage-based biocontrol agent.

## 2. Materials and Methods

### 2.1. Phages and Bacterial Strains

Bacteriophage AAS23 was originally isolated from the outwash of jostabeere berries collected in Lithuania. *Pantoea agglomerans* strain AUR was used as the host for AAS23 isolation, propagation and phage growth experiments. The bacterial strains used in this study for host range determination are listed in Table S2 [27,28]. All bacteria and phages were cultivated in Luria–Bertani broth (LB) or LB agar.

### 2.2. Phage Isolation, Propagation and Purification Techniques

Phage isolation was performed by using the enrichment of phages in the source material technique as described previously [13], using a local isolate, *Pantoea agglomerans* strain AUR, as a host. Phage titration was performed by using the soft agar overlay method described by Adams [29], with minor modifications. Briefly, 0.1 mL of diluted phage suspension was mixed with 0.5 mL of indicator cells (OD_600_–0.5). The mixture then was added to 2.5 mL of 0.4% (w/v) soft agar and poured over the 1.2% LB agar plate as a uniform layer. The plates were incubated 1–10 days at 3–40°C before the enumeration and measurement of plaques. For comparison of the phage and bacterial counts in the different zones (lysis, halo and bacterial zone), an equally large surface of the top agar was removed from each zone, suspended in 0.1 mL of LB-medium and vortexed. Plaque forming units were determined using the double agar method. Initial AAS23 purification was performed by five consecutive transfers of phage from individual plaques to new bacterial cell lawns. Notably, as AAS23 propagated poorly in a liquid broth, the propagation of phage was performed by the soft agar overlay method, as described previously [30], using *Pantoea agglomerans* strain AUR as a host. The phage purification was performed using a CsCl step gradient [31], as described by Šimoliūnas et al. [28]. The adsorption tests were carried out as described by Kropinski [32]. For determination of the efficiency of plating (EOP), high-titer phage stocks were diluted and plated in triplicate. Plates incubated at 3–40 °C were read after 1–10 days of incubation. The temperature at which the largest number of plaques formed was taken as the standard for the EOP calculation.

### 2.3. Transmission Electron Microscopy

The CsCl density gradient-purified phage particles were diluted to approximately 10^11^ PFU/mL with distilled water, 10 µL of the sample was directly applied on the carbon-coated nickel grid (Agar Scientific, Essex, UK), drained with filter paper, stained with two successive drops of 2% uranyl acetate (pH 4.5), dried, and examined in Tecnai G2 F20 X-TWIN transmission electron microscope (FEI, Hillsboro, OR, USA).

### 2.4. DNA Isolation and Restriction Analysis

The aliquot of high-titer (10^11^–10^12^ PFU/mL) phage suspension was subjected to phenol/chloroform extraction and ethanol precipitation, as described by Carlson and Miller [33]. The isolated phage DNA was subsequently used for PCR and restriction analysis, or it was subjected to genome sequencing. The restriction digestion was performed with Bsu15I, Csp6I, DraI, EcoRII, EcoRV, HhaI, MboI and NdeI restriction endonucleases (Thermo Fisher Scientific, Vilnius, Lithuania), according to the supplier’s recommendations. The DNA fragments were separated by electrophoresis in a 0.8% agarose gel containing ethidium bromide. A restriction analysis was performed in triplicate to confirm the results.

### 2.5. Genome Sequencing and Analysis

The complete genome sequence of AAS23 was determined using Illumina DNA sequencing technology at BaseClear, in the Netherlands. Single-end or paired-end sequence reads were generated using the Illumina NovaSeq 6000 or MiSeq system. The sequences generated with the MiSeq system were performed under accreditation according to the scope of BaseClear B.V. (L457; NEN-EN-ISO/IEC 17025).

When paired-end sequencing is being performed, the “Number of reads” noted in this report is referring to read pairs. FASTQ read sequence files were generated using bcl2fastq version 2.20 (Illumina). Initial quality assessment was based on data passing the Illumina Chastity filtering. Subsequently, reads containing PhiX control signal were removed using an inhouse filtering protocol. In addition, reads containing (partial) adapters were clipped (up to a minimum read length of 50 bp). The second quality assessment was based on the remaining reads using the FASTQC quality control tool version 0.11.5 (https://www.bioinformatics.babraham.ac.uk/projects/fastqc/, accessed on 11 December 2020). The quality of Illumina reads was improved using the error correction tool BayesHammer [34]. Error-corrected reads were assembled into contigs using SPAdes version 3.10 [35]. The order of contigs, and the distances between them, were estimated using the insert size information derived from an alignment of the paired-end reads to the draft assembly. Consequently, contigs were linked together and placed into scaffolds using SSPACE version 2.3 [36]. Using Illumina reads, gapped regions within scaffolds were (partially) closed using GapFiller version 1.10 [37]. Finally, assembly errors and the nucleotide disagreements between the Illumina reads and scaffold sequences were corrected using Pilon version 1.21 [38]. Thus, the reads of AAS23 were assembled into a single linear contig of 51,247 bp (134,433 mapped reads; 383.86 average coverage). The ends of the contig were confirmed using PCR, followed by Sanger sequencing reactions at Macrogen (Seoul, South Korea). PCR fragments were obtained by the amplification of AAS23 phage wild-type DNA using AAS23_F1 5′-AGATCGAAACCATCACCAATG-3′ and AAS23_R1 5′-GTCAGAGGACGATAAACAATG-3′ primers. In order to determine the termini of the genome of AAS23, restriction analysis was performed based on recommendations by Casjens and Gilcrease [39]. No defined genomic termini were identified, and to preserve gene contiguity, the genome start point was selected from the predicted terminase small subunit gene.

The open reading frames (ORFs) were predicted with Geneious Prime 2020 (http://www.geneious.com, accessed on 15 December 2020), using a minimum ORF size of 60 nt. The analysis of genome sequence was performed using the Fasta-Protein, Fasta-Nucleotide, BLASTP, Transeq (http://www.ebi.ac.uk/Tools/st/emboss_transeq, accessed on 10 February 2021), and Clustal Omega (http://www.ebi.ac.uk/Tools/msa/clustalo, accessed on 10 February 2021), as well as HHpred, HHblits, HMMER, and HHsenser [40,41]. ARAGORN (http://130.235.244.92/ARAGORN, accessed on 18 December 2020) and tRNAscan-SE 1.21 (http://lowelab.ucsc.edu/tRNAscan-SE, accessed on 18 December 2020) were used to search for tRNAs. Phylogenetic analysis was conducted using MEGA version 7 [42] and ViPTree was used for the total proteome comparisons [43]. The overall nucleotide sequence identity was calculated using PASC [44].

### 2.6. Analysis of Structural Proteins

An analysis of the structural proteins of AAS23 virions was performed using a modified filter-aided sample preparation (FASP) protocol, followed by LC-MS/MS analysis, as described previously [30].

### 2.7. Nucleotide Sequence Accession Numbers

The complete genome sequence of the *Pantoea* bacteriophage AAS23 was deposited in the EMBL nucleotide sequence database under accession number MK095606.

## 3. Results

### 3.1. Phage Morphology, Host Range, and Physiological Characteristics

**Figure 1:**
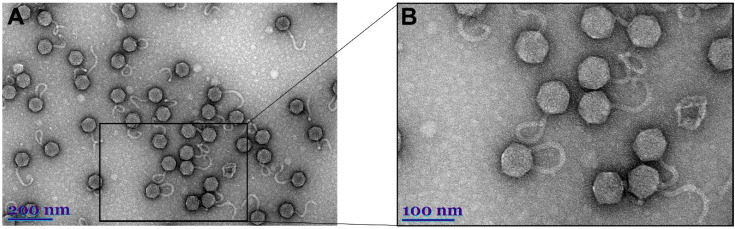
Electron micrographs of vB_PagS_AAS23 virions.

Transmission electron microscopy (TEM) of AAS23 virions revealed siphovirus that fits the B1 morphotype in Bradley’s classification [45,46]. Phage AAS23 is characterized by an isometric head (66.21 ± 2.08 (*n* = 28) nm in diameter) and an apparently non-contractile flexible tail (153.15 ± 10.66 (*n* = 20) nm in length, 11.19 ± 1.58 (*n* = 24) in width) (Figure 1). Although no tail fibers were clearly visible by TEM, the genes coding for the tail fiber components were detected by bioinformatics analysis of the genome and/or by proteomics of phage virions (see below).

**Figure 2:**
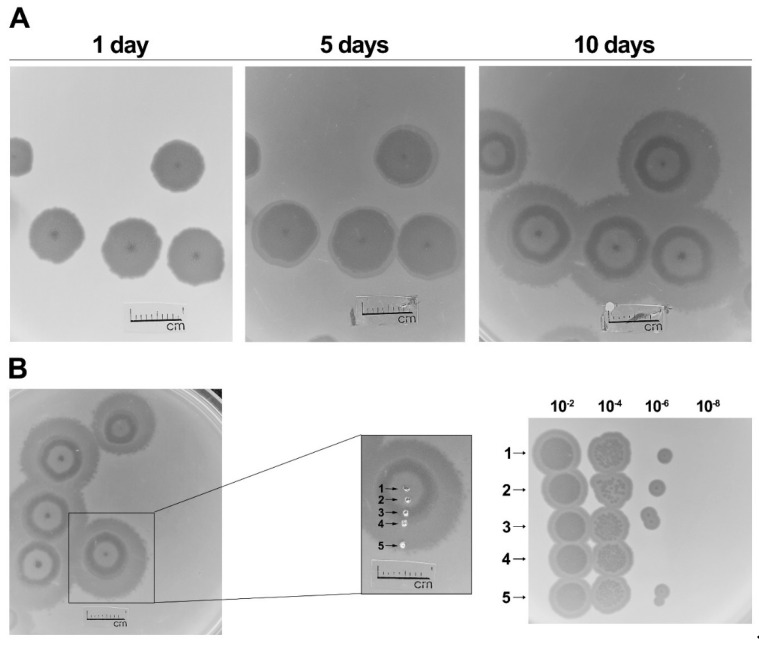
Morphology of plaques formed by vB_PagS_AAS23 on a lawn of *Pantoea agglomerans* strain AUR, and comparison of AAS23 count in the different plaque zones. (**A**) Plates were incubated at 22 °C, numbers above indicate days of incubation. (**B**) Plates were incubated at 22 °C for 10 days; the numbered rows indicate sampling sites in the different zones, and values at the top indicate dilution series.

In total, 24 bacterial strains (Table S2) were used to explore the host range of bacteriophage AAS23. With an exception of *Pantoea agglomerans* strain AUR, the other tested *Pantoea* spp. isolates, as well as all of the tested strains of *Acinetobacter*, *Citrobacter*, *Erwinia*, *Escherichia*, *Klebsiella*, *Salmonella*, and *Pseudomonas* spp. were found to be resistant to AAS23. In order to determine the optimal conditions for phage propagation, the effect of temperature on the efficiency of plating (e.o.p.) was examined in the temperature range of 3–40 °C. The test revealed that AAS23 is a cold-adapted virus—it forms plaques at 4–32 °C, and has an optimum temperature for plating at about 22 °C (Figure S1). After 24 h of incubation at 22 °C, AAS23 forms plaques with a clear center and turbid edge (up to 10.1 ± 0.78 mm in diameter). The phage produces plaques surrounded by a constantly growing opaque halo zones (Figure 2). After an incubation at 22 °C for five days, the plaques of AAS23 reach 12.23 ± 0.39 mm in diameter, and the plaques further increase in diameter (20.48 ± 0.53 mm) within a period of 10 days.

According to the literature, the halo zones surrounding the phage plaques indicate the presence of phage-encoded bacterial exopolysaccharide (EPS)-degrading depolymerases, which act either as integral components of the virion particles or as free soluble enzymes [47]. Within the halo zone, both phage particles and viable bacteria are often found, and it has been suggested that halo formation is not only caused by the excess of EPS depolymerases produced inside the host during phage replication and released after cell lysis, but also by viral diffusion in the bacterial lawn from the primarily infected cell [48,49]. For comparison of AAS23 counts in the different plaque zones, phage enumeration of equally large surfaces of the lysis, halo and bacterial zones was performed (Figure 2). This test demonstrated that all of the five sampled zones harbored almost equal amounts of the infective phage particles (Figure 2B), while phages were completely absent in the outside bacterial zone (data not shown). Thus, it is likely that the formation of halo zones in case of AAS23 may be related to effective diffusion of virions out of primarily infected cells.

The attempts to obtain a one-step growth curve of AAS23 were unsuccessful because of the slow phage adsorption kinetics. Under optimal conditions determined for phage growth, only about 55% of the input virions adsorbed to the host cells during first 5 min, and, as many as 20% of the input virions remained unattached after 20 min (Figure S2).

### 3.2. Overview of Genome

**Figure 3:**
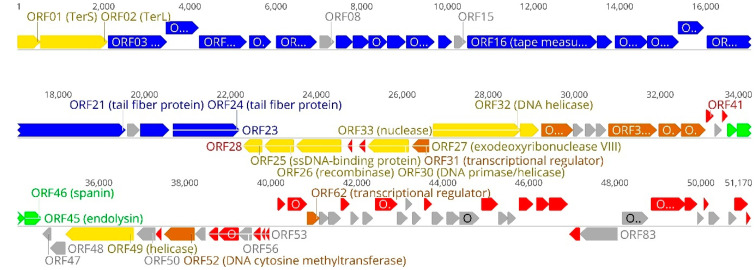
Functional genome map of bacteriophage vB_PagS_AAS23. The coding capacity of the genome is shown. Numbers indicate open reading frame (ORF) position in genome, functions are assigned according to the characterized ORFs in NCBI database and HHpred analysis. The color code is as follows: yellow—DNA replication, recombination, repair and packaging; blue—structural proteins; brown—transcription, translation, nucleotide metabolism; green—lysis, phage-host interaction; grey—conserved hypothetical proteins; red—AAS23 hypothetical proteins with no reliable identity to database entries.

Phage AAS23 has a linear, double stranded DNA genome (51,170 bp) with a G–C content of 47.6%, which is insignificantly lower to that (52–55%) observed for *Pantoea* spp. [1]. The results of the PCR and restriction-digestion analyses suggest that genome of AAS23 is a circularly permuted molecule. Similar to other dsDNA bacteriophages, the genome of AAS23 is close-packed-93.8% of the genome is coding. Based on the results of bioinformatics analysis, it contains 92 probable protein-encoding genes, and no genes for tRNA (Figure 3). Notably, an apparent asymmetry in the distribution of the genes on the two DNA strands was observed. In total, 70 ORFs of AAS23 are predicted to be transcribed from the same DNA strand, while the other 22 ORFs are found on the opposite DNA strand.

Bioinformatics analysis revealed that 25 out of 92 AAS23 ORFs encode unique proteins that have no reliable identity (E-values > 0.001) to the database entries. The ORFs that encode proteins with matches to those in other sequenced genomes, the percentage of amino acid identity ranges from 29% to 81% and, in most cases (46 out of 67 ORFs), from 50% to 75% (Table S3). Among the AAS23 gene products with detectable homologs in other sequenced genomes, 46 have the best E-values to proteins from phages that infect *Cronobacter, Enterobacter*, *Erwinia, Escherichia*, *Klebsiella*, *Pantoea, Salmonella* and *Xanthomonas*. Two AAS23 gene products share similarity to proteins found in bacteria exclusively: putative transcriptional regulator encoded by ORF62 has the best match with hypothetical protein (WP_071882931.1) from *Pantoea* sp. PSNIH1, whereas the amino acid sequence of a hypothetical protein encoded by ORF78 shares the highest identity with hypothetical protein (WP_010281918.1) from *Pectobacterium brasiliense* (Table S3).

Based on homology to biologically defined proteins, 38 ORFs of AAS23 were given a putative functional annotation (Table S3). As was observed in other siphoviruses, genome of AAS23 appears to have a modular organization, with genes for DNA packaging, structure/morphogenesis, host lysis, replication/regulation and nucleotide metabolism clustered together (Figure 3). Notably, none of the predicted gene products shows sequence homology with integration-related proteins, antibiotic resistance determinants, or virulence factors.

### 3.3. Structural Proteins

Bioinformatics analysis of the AAS23 genome sequence allowed the identification of 18 structural genes, including those coding for head (ORF03–ORF07, ORF09–ORF10), tail (ORF11–ORF14, ORF16–ORF20) and tail fiber (ORF21, ORF24) proteins (Table S3). The major capsid protein (MCP) and major tail protein (MTP) are two of the main building blocks constituting the virions of siphoviruses [50]. Bioinformatics analysis revealed that major capsid protein (gp07) of AAS23 belongs to DUF2184 (cl21556) superfamily and exhibits the highest similarity to major capsid proteins from a variety of Enterobacteria-infecting bacteriophages. HHpred analysis revealed that AAS23 gp07 best matches the structure of the major capsid protein (gp57) of *Pseudoalteromonas* phage TW1 (5WK1_G; E-value, 4.3 × 10^−34^). The major tail protein (gp13) of AAS23 belongs to Phage_tail_3 (cl07426) superfamily and has the best HHpred hit to the major tail protein (gp5) of Enterobacteria phage lambda (2K4Q_A; probability, 99.61%; E-value, 3.3 × 10^−14^).

As mentioned above, although no tail fibers were clearly visible by TEM (Figure 1), two AAS23 genes coding for tail fiber proteins (gp21 and gp24) have been identified by bioinformatics approaches. The tail fiber protein encoded by ORF21 contains conserved COG4733 (COG4733), Smc (COG1196), and DUF1983 (pfam09327) domains, whereas the tail fiber protein encoded by ORF24 has a C-terminal Peptidase_S74 domain (pfam13884), a conserved chaperone domain that is commonly found in endosialidases. The hypothetical protein (gp22) and the putative structural protein (gp23) are inserted between the tail fiber proteins of AAS23 (Figure 3). No conserved domains have been identified in these two proteins, but gp22 and gp23 share the best homology with the gp23 and gp24 of *Erwinia* phage vB_EhrS_49, as well as the gp29 and gp30 of *Erwinia* phage Midgardsormr38, respectively. These genes of *Erwinia* phages are located just downstream the host specificity protein J (gp22 and gp28, respectively). Thus, based on the position in the genome, homology to publicly available proteins, and results of proteomics analysis, it is likely that gp22, gp23 of AAS23, and their homologues in *Erwinia*-infecting phages, may play important role in phage-host interactions.

FASP followed by LC-MS/MS confirmed that all of the aforementioned structural proteins, except for gp09 (head-tail adaptor), gp10 (head completion protein), gp12 (tail completion protein), gp14 (tail assembly chaperone), and gp19 (tail-associated protein), are present in the virion of AAS23 (Table S4). Indetermination of potential structural proteins, which were identified by bioinformatics approaches but not detected by proteomics analysis might be due to the incompatibility of these proteins with sample preparation procedures or/and because of their low abundance in virions. On the other hand, FASP followed by LC-MS/MS led to the experimental identification of phosphoesterase (gp38) and two proteins, gp23 and gp83, which share similarity exclusively to hypothetical proteins. In addition, AAS23 gp82, which has no reliable homology to any entries in the public databases to date, was also detected, suggesting that it may be a virion-associated protein. As seen in Figure 3, all of AAS23 structural genes are found within a large genome cluster (~20 kb) located just downstream the packaging modules.

### 3.4. Packaging

The packaging machine of tailed bacteriophages usually consists of two essential components: a portal ring and a terminase complex [51]. Most characterized terminases consist of a small subunit (TerS) involved in DNA recognition and a large terminase subunit (TerL) containing the ATPase and the endonuclease activities [52]. AAS23 ORF03 was annotated as a portal protein based on the similarity to the corresponding proteins of various bacteriophages and was detected by proteomics approaches as well (Figure 3; Table S4). The gp03 of AAS23 contains conserved DUF1073 (pfam06381) domain and has the best HHpred match with portal protein of *Rhodobacter capsulatus* (6TE9; HHpred probability, 99.94%; E-value, 1.8 × 10^−24^). The TerS and TerL of AAS23 are encoded by ORF01 and ORF02, respectively, and share homology to TerS and TerL from a wide range of diverse phages. The AAS23 TerS have been predicted to belong to the GP3_package (pfam16677) superfamily and shows the highest similarity to the terminase small subunit of Enterobacteria phage P22 (3P9A_G; HHpred probability, 99.97%; E-value, 1.9 × 10^−30^). The AAS23 TerL contains two conserved domains: COG5410 (E-value, 1.27 × 10^−52^) of Terminase_6 superfamily is presented in the N terminus (aa 11 to 317) whereas psiM2_ORF9 (E-value, 1.88 × 10^−23^) is detectable in the C terminus (aa 360 to 500) of MED16 gp02. HHpred yielded the best hit to the terL of Enterobacteria phage T7 (4BIJ_E; probability, 100%; E-value, 4.1 × 10^−36^).

### 3.5. DNA RRR

Bioinformatics analysis revealed a set of AAS23 genes associated with DNA replication, recombination, and repair (DNA RRR) that, unlike structural genes, are apparently scattered throughout the genome. However, the genome of AAS23 contains no homologues to characterized DNA polymerase genes, suggesting that this phage most likely uses DNA polymerase of the host cell.

The product encoded by ORF25 of AAS23 was identified as a single-stranded DNA binding (SSB) protein. The SSB proteins are ubiquitous within all kingdoms of life and in DNA viruses. They protect ssDNA intermediates during replication, repair and recombination [53]. The SSB protein of AAS23 contains conserved PRK09010 domain of RPA_2b-aaRSs_OBF_like superfamily (cl09930) and DUF3127 domain of DUF3127 superfamily (cl12864). The gp25 of AAS23 shows similarity to SSB proteins from a variety of organisms including bacteriophages and has the best HHpred match with a single-stranded DNA-binding protein of *Escherichia coli* K-12 (4MZ9_D; probability, 99.9%; E-value, 5.4 × 10^−22^).

The RRR proteins of AAS23 include a number of helicases - enzymes playing essential role in dsDNA strand separation. The ORF32 encodes DNA helicase containing a conserved SSL2 domain of SSL2 superfamily (cl34083). The gp30 is a DNA primase/helicase with a conserved Prim_Zn_Ribbon (E-value, 8.82 × 10^−14^) and COG4643 (E-value, 8.82 × 10^−13^) domains of zf-CHC2 (cl21601) and COG4643 (cl26703) superfamilies, respectively. The gp49 of AAS23 has been identified as putative helicase containing a conserved DUF3987 domain (E-value, 4.70 × 10^−37^) of DUF3987 superfamily (cl20483). Additionally, bioinformatics analysis allowed identification of the phage-encoded VRR-NUC superfamily (cl22959) nuclease, which shares homology to VRR_NUC domain-containing nuclease of *Salmonella* phage SETP3 (4QBL_F; HHpred probability 99.77%; E-value, 1.4 × 10^−17^). Other AAS23 gene products possibly involved in DNA recombination are recombinase (gp26) containing an ERF domain (pfam04404) in its N terminus and exodeoxyribonuclease VIII (gp27) containing the C-terminal DUF3799 domain (pfam12684).

### 3.6. Nucleotide Metabolism and DNA Modification

Based on the amino acid sequence similarity, a set of AAS23 gene products, the phosphoesterase, 3’-phosphatase/5’-PNK and deoxynucleoside monophosphate kinase encoded by ORF38, ORF39 and ORF40, respectively, were assigned as nucleotide metabolism enzymes. The gp38, gp39 and gp40 of AAS23 share the highest homology with the hypothetical protein AXI63_gp63 of *Klebsiella* phage KP36 (70% aa identity; E-value 0.0), 3’-phosphatase, 5’-polynucleotide kinase of *Enterobacter* phage Ec_L1 (75% aa identity; E-value 2.0 × 10^−79^) and putative ATP-binding protein of *Escherichia* phage Henu7 (54% aa identity; E-value 1.0 × 10^−58^), respectively.

To protect the genomic DNA from restriction endonucleases of the host cell, phages employ various strategies, including adenine and cytosine methylation [54]. Based on the results of bioinformatics analysis, two putative methylases are present in the genome of AAS23. The Dam superfamily (cl05442) DNA *N*^6^-adenine-methyltransferases is encoded by ORF34, whereas the gene product of AAS23 ORF52 was identified as DNA cytosine methyltransferase. Although no conserved domains were identified of AAS23 gp52 by BLASTP analysis, HHpred yielded the best hits to a number of methylases including cytosine-specific methyltransferase of *Shigella flexneri* (3ME5_A; HHPred probability, 99.7%; E-value, 8.2 × 10^−18^). The restriction digestion analysis (Figure S3) revealed that the DNA of AAS23 is Dam and Dcm methylated, suggesting that DNA *N*^6^-adenine-methyltransferase and DNA cytosine methyltransferase of AAS23 ORF52 are functional enzymes.

### 3.7. Lysis Cassette

All dsDNA phages accomplish host lysis using a muralytic enzyme (known as an endolysin) and a holin, a small membrane protein that permeabilizes the membrane at a programmed time [55], whereas Gram-negative hosts-infecting phages also use spanins, which are required to disrupt the outer membrane [56]. The lysis cassette of phage AAS23 encodes all three lysis proteins in the canonical order: holin (gp44), endolysin (gp45), and spanin (gp46) (Figure 3). All predicted AAS23 lysis proteins show similarity to appropriate lysis proteins from a variety of phages. AAS23 endolysin has been predicted to belong to the Lyz-like superfamily (cl00222), whereas spanin is a unimolecular spanin (u-spanin). Unlike the two-component spanins encoded by many of the other phages, including lambda, the unimolecular spanins are not so frequent and have not been studied extensively yet [57].

### 3.8. Phylogenetic Analysis

**Figure 4:**
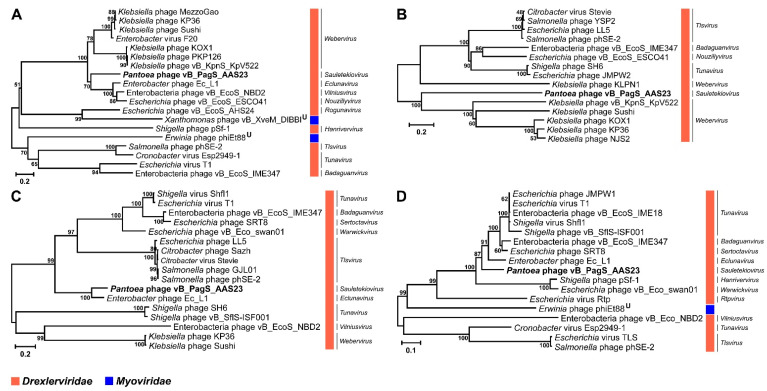
Neighbor-joining tree analysis based on the alignment of the amino acid sequences of *Pantoea* phage vB_PagS_AAS23: (**A**) major capsid protein, (**B**) tape measure protein (TMP), (**C**) helicase, and (**D**) terminase large subunit (terL). The percentage of replicate trees in which the associated taxa clustered together in the bootstrap test is shown next to the branches.

In order to determine the phylogenetic relationship between AAS23 and its closest relatives to date, the comparison of the individual genes most often used for the analysis of the evolutionary relationships between bacteriophages [58] was carried out. The phylogenetic trees based on the alignment of the AAS23 major capsid protein, tape measure protein, helicase and terminase large subunit aa sequences with those returned by BLASTP homology searches were constructed (Figure 4). All the phylogenetic trees showed that AAS23 is phylogenetically distant from other phages and represents an evolutionarily distinct branch of siphoviruses. As seen in Figure 4, AAS23 occupies a somewhat intermediate position between siphophages belonging to the genera *Eclunavirus*, *Webervirus*, *Tlsvirus*, *Warwickvirus*, *and Tunavirus* within the family *Drexlerviridae*.

**Figure 5:**
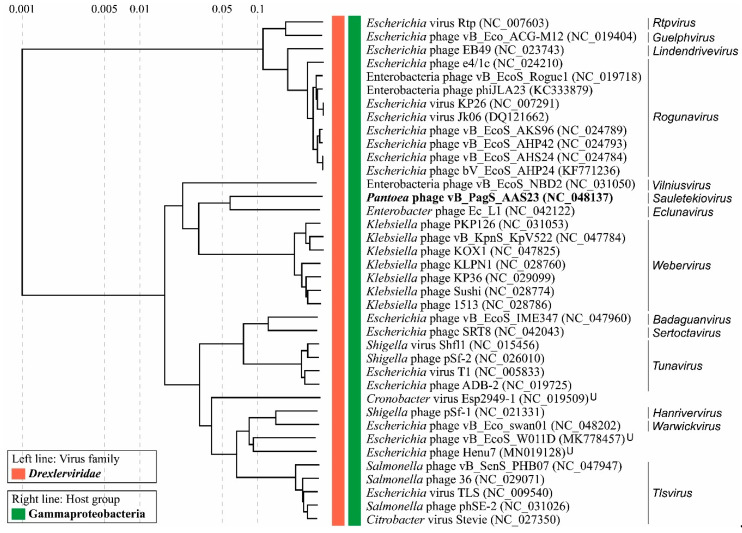
ViPTree generated proteomic tree of *Pantoea* phage vB_PagS_AAS23 and dsDNA viruses represented in the rectangular view. The tree is constructed by BIONJ based on genomic distance matrixes, and mid-point rooted. Branch lengths are logarithmically scaled from the root of the entire proteomic tree. The numbers at the top represent the log-scaled branch lengths based on the SG (normalized tBLASTx scores) values.

To obtain a more detailed picture of the phylogenetic relationships of AAS23 and other viruses, the overall nucleotide sequence identity was calculated using PASC, and a comparative total proteome comparison was performed using ViPTree. Based on the whole-proteome alignment of AAS23 and its closest relatives analyzed, it was confirmed that this phage represents a distinct branch on the neighbor-joining tree (Figure 5). Additionally, AAS23 is the closest to *Enterobacter* phage Ec_L1 - a single species of the genus *Eclunavirus*.

**Figure 6:**
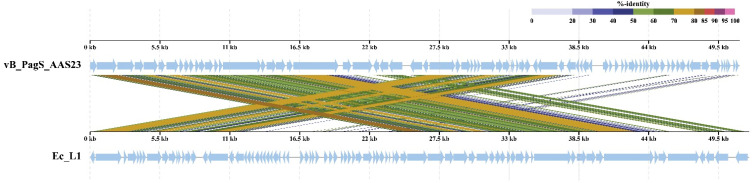
ViPTree generated whole-proteome alignment of *Pantoea* phage vB_PagS_AAS23 and *Enterobacter* phage Ec_L1. Colored lines in the alignment indicate tBLASTx results (E-value < 0.01). Positions of each sequence are automatically adjusted (i.e., circularly permuted and reverse stranded) for clear representation of collinearity between genomes.

In order to determine the most homologous regions in the genomes of AAS23 and Ec_L1, genome alignment was performed by using ViPTree. Genomes of both bacteriophages share several regions of nucleotide similarity that, in AAS23 and Ec_L1, cover the essential structural and virion morphogenesis proteins-encoding genes, as well as genes related to DNA metabolism and modification (Figure 6). Nevertheless, the overall nucleotide sequence identity between AAS23 and its closest relatives are quite low, and ranges from 46.58% (AAS23 vs. *Enterobacter* phage Ec_L1) to 40.08% (AAS23 vs. *Escherichia* phage vB_EcoS_swan01) (Table S5).

## 4. Discussion

In 2019, it was proposed to create a new genus *Sauletekiovirus* within the family *Drexlerviridae* containing a single species, *Pantoea* phage AAS23 (https://talk.ictvonline.org/taxonomy/p/taxonomy-history?taxnode_id=201908174, accessed on 20 February 2021). In this study, we present a detailed analysis of AAS23 phage genome, virion morphology and proteomics, and other characteristics that are relevant for comprehensive description of bacteriophages. The results of the comparison of the individual genes, a total proteome and overall nucleotide identity demonstrate that AAS23 has no close phylogenetic relatives known so far. It was demonstrated that AAS23 shares the highest overall nucleotide sequence identity (46.58%) with Enterobacter phage Ec_L1. According to the Bacterial and Archaeal Viruses Subcommittee (BAVS) of the ICTV, a genus is described as a cohesive group of viruses containing a high degree (>50%) of nucleotide sequence similarity [59]. Following this, and, based on the comparative genome sequence analysis performed during this study, bacteriophage AAS23 is a single species of the genus *Sauletekiovirus* within the family *Drexlerviridae* to date.

The genome (51,170 bp) of AAS23 contains no homologues to characterized DNA polymerase genes, suggesting that this phage most likely uses DNA polymerase of the host cell. According to the literature, a clear correlation between the size of the genome and self-sufficiency of viral DNA replication exists. Bacteriophages with large genomes (>140 kb) usually encode their own replication machinery, whereas viruses with a relatively small genomes (<40 kb) tend to employ a replicative polymerase of the host cell [60]. Such “less self-sufficient” dsDNA bacteriophages usually encode a set of proteins, that all play principal roles in the recruitment of the host DNA replication machinery. For example, it was demonstrated that phage-encoded helicase, helicase loader, origin binding protein (OBP) and single-stranded DNA-binding protein of *Bacillus subtilis* phage SPP1 are vital proteins to achieve efficient viral replication [61]. Although no genes for a helicase loader or OBP were detected in the genome of AAS23, a large number of the genes for putative proteins of unknown function surround those AAS23 RRR genes that have been identified by bioinformatics approaches. It is possible that the genome of this siphovirus encodes a yet-unrecognized helicase loader or OBP. On the other hand, Costa and colleagues suggested that replicative helicases could also be involved in the recognition of replication origins [62]. Taking into account that AAS23 encodes even three helicases, there is a possibility that AAS23 uses its helicase/helicases and SSB protein complex to recruit replicative DNA polymerase of the host cell.

Phage AAS23 was isolated from the outwash of jostabeere berries using a local isolate, *Pantoea agglomerans* strain AUR, as a host. According to Kering and coauthors [63], isolation of phages from the phyllosphere is highly desirable as they could be adapted to that environment for control of phytopathogens. It seems that phage AAS23 could be a good example of such adaptation, because it is capable of replicating at ambient temperature (Figure S1) and forms plaques surrounded by opaque halo zones with an increasing diameter over the course of time. Almost sixty years ago, those halo zones have been already described as an indicator of the phage-associated EPS depolymerases, the enzymes capable of degrading bacterial polysaccharides [64]. Today, phage-encoded depolymerases became a promising alternative for detection and control of bacterial pathogens in agriculture, food industry, and medicine [65]. The vast majority of these depolymerases are encoded by genes for the structural proteins (mostly in the tail fibers, baseplate, and sometimes in the neck) or in their close proximity, and thus are considered as structural proteins [47,65,66,67]. The AAS23-encoded depolymerase is predicted in the tail fiber protein (gp24) containing C-terminal Peptidase_S74 domain (aa 402 to 459), which has been found in those phage-derived depolymerases that hydrolyze capsular polysaccharides with sialic acid. The sialidases containing Peptidase_S74 domain are present in a wide range of phages targeting different bacterial species but no Peptidase_S74 domain-containing sialidases have been detected in *Pantoea* or *Erwinia* phages to date [47].

During this study we examined that Peptidase_S74 domains are present in the tail fiber proteins gp20, gp53 and gp26 of *Pantoea* phages vB_PagS_AAS21, Phynn, and *Erwinia* phage vB_EhrS_49, respectively. However, bioinformatics analysis revealed that the AAS23-encoded depolymerase (gp24) shares only a low aa identity with the aforementioned tail fiber proteins of *Pantoea* and *Erwinia* phages, as well as with other homologues from NCBI database. In order to determine the phylogenetic relationship between AAS23 gp24 and its closest relatives, a phylogenetic tree based on the alignment of the gp24 (as well as Peptidase_S74 domain of gp24) aa sequences with those returned by BLASTP homology searches was constructed (Figures S4,S5). This analysis demonstrates that neither AAS23 gp24 nor Peptidase_S74 domain of gp24 have close homologues to those of other phages. Thus, gp24 and Peptidase_S74 domain of gp24 have an interest for further investigation.

A number of studies have demonstrated the feasibility of applying phages as biocontrol agents against the phytopathogens [68,69,70]. *Inter alia*, some promising results have been achieved by using phages in preventing or controlling the fire blight causing agent - *Erwinia amylowora* [18,20,71]. In contrast, although it has been reported that the bacteria from the genus *Pantoea*, a close relative of *Erwinia*, also cause the diseases of cultivable plants, as well as opportunistic human infections [7], only a limited amount of information on *Pantoea*-infecting bacteriophages is known to date. Thus, it is very important that viruses, which have a potential as biocontrol agents, must be examined in detail particularly regarding a presence of the undesirable genes. No genes encoding integration-related proteins, antibiotic resistance determinants, or virulence factors are identified in the genome of *Pantoea agglomerans*-infecting phage AAS23. From a practical point of view, it is important that bacteriophage AAS23 is a cold-adapted lytic siphovirus demonstrating depolymerase activity, and is able to replicate even at 4 °C. Thus, the results of this study not only suggest that AAS23 or its gene products have a potential to be used as biocontrol agents, but also provide new insights that deepen our understanding of *Pantoea* phages.

## Data Availability

The data presented in this study are openly available in the GenBank repository.

## Supplementary Materials

The following are available online at https://www.mdpi.com/2076-2607/9/3/668/s1. Table S1: A list of *Pantoea* bacteriophages with completely sequenced genomes, which have been published and/or deposited in the public databases, Table S2: Bacterial strains used in this study to determine the host range of phage AAS23, Table S3: AAS23 ORFs-having homologues with reliable identity (E-values >0.001) in other viruses or cellular organisms, Table S4: Structural AAS23 proteins identified by MS, Table S5: Top matches for BLAST-based alignments of whole genome sequences of AAS23 and its closest relatives generated using PASC, Figure S1: Effect of temperature on the plating efficiency of phage AAS23, Figure S2: Adsorption rate of phage AAS23, Figure S3: Restriction digestion patterns of AAS23 genomic DNA, Figure S4: Neighbor-joining tree analysis based on the alignment of the amino acid sequences of AAS23 tail fiber protein (gp24) and its closest homologues, Figure S5: Neighbor-joining tree analysis based on the alignment of the amino acid sequences of conserved Peptidase_S74 domain (aa 402 to 459) of AAS23 tail fiber protein (gp24) and its closest homologues.
