# Supplementary material for: Pantoea Bacteriophage vB_PagS_AAS23: A Singleton of the Genus Sauletekiovirus
Source: Microorganisms. 2021 Mar 23;9(3):668. doi: 10.3390/microorganisms9030668 (PMC8004638; doi:10.3390/microorganisms9030668)
Supplement: Supplementary file 1 [file microorganisms-09-00668-s001.zip › Supplementary material/Microoganisms Suppl.pdf]

Table S1. A list of *Pantoea* bacteriophages with completely sequenced genomes, which have been published and/or deposited in the public databases.

Phage	GenBank accession no.	Family	Genome size (bp)	Reference
Phynn	MN038175.1	*Myoviridae*	173720	unpublished
vB_PagM_LIET2	MK388689.1	*Myoviridae*	74710	unpublished
Kyle	NC048796.1	*Myoviridae*	73168	unpublished
vB_PagM_SSEM1	MT230534.1	*Myoviridae*	54982	unpublished
vB_PagM_AAM37	MK798143.1	*Myoviridae*	49990	unpublished
vB_PagM_PSKM	MK798144.1	*Myoviridae*	49935	unpublished
vB_PagM_AAM22	MK798142.1	*Myoviridae*	49744	[11]
vB_PagS_AAS21	MK770119.1	*Siphoviridae*	116649	[12]
vB_PagS_Vid5	MG948468.1	*Siphoviridae*	61437	[13]
**vB_PagS_AAS23**	**MK095606.1**	***Siphoviridae***	**51170**	**[this study]**
vB_PagS_MED16	MK095605.1	*Siphoviridae*	46103	unpublished
LIMElight	FR687252.1	*Podoviridae*	44546	[14]
LIMEzero	FR751545.1	*Podoviridae*	43032	[14]
vB_PagP-SK1	MN450150.1	*Podoviridae*	39938	[15]

Table S2. Bacterial strains used in this study to determine the host range of phage AAS23.

Strain	Relevant characteristics	Source or reference
Acinetobacter baumannii #46		Prof. E. Sužiedėlienė
Citrobacter freundii		Prof. E. Sužiedėlienė
Erwinia carotovora 8982		Prof. E. Sužiedėlienė
Erwinia carotovora 961–63		Prof. E. Sužiedėlienė
Erwinia piriflorinigrans 26166	type strain	DSMZ
Erwinia billingiae 17872	type strain	DSMZ
Escherichia coli B40	*sup*D	Dr. L. W. Black
Escherichia coli B^E	sup^0	Dr. L. W. Black
Escherichia coli BL21	F^- *dcm ompT hsdS* (rB^-mB^-) *gal*	Novagen
Escherichia coli BW25113	[Δ(*araD–araB*)*567* Δ(*rhaD–rhaB*)*568 ΔlacZ4787* (::rrnB-3) *hsdR514 rph-1*]	[27]
Escherichia coli DH10B	F^- *endA1 recA1 galE15 galK16 nupG rpsL* Δ*lacX74* Φ80*lacZ*ΔM15 *araD139* Δ(*ara,leu*)7697 *mcrA* Δ(*mrr-hsdRMS-mcrBC*) λ^-	Invitrogen
Escherichia coli MG1655	F^- lambda$^-$ *ilvG*$^-$ *rfb*-50 *rph*-1	Prof. E. Sužiedėlienė
Escherichia coli MH1	*araD139* Δ*lacX74 galU galK hsr hsm rpsL*	Dr. K. N. Kreuzer
Klebsiella sp. KV-3	Veterinary isolate, Amp^r, Str^r, Tet^r, Kan^s, Gm^s, Nc^s,$Cl^{r/s}$	[28]
Pantoea agglomerans ARC	environmental isolate	[13]
Pantoea agglomerans AUR[a]	environmental isolate	[13]
Pantoea agglomerans BSL	environmental isolate	[13]
Pantoea agglomerans DDM	environmental isolate	[13]
Pantoea agglomerans MMG	environmental isolate	[13]
Pantoea agglomerans SER	environmental isolate	[13]
Pantoea agglomerans 3493	type strain	DSMZ
Pantoea conspicua 24241	type strain	DSMZ
Pseudomonas aeruginosa PAO1		Prof. E. Sužiedėlienė
Salmonella enterica ser. Typhimurium 292		Prof. E. Sužiedėlienė

a – AAS23-sensitive strain.

Table S3. AAS23 ORFs-having homologues with reliable identity (E-values >0.001) in other viruses or cellular organisms.

AAS23 ORF (position)	Predicted function	Significant match	Identity aa %/ similarity aa% (length of the overlapping segment)	E value
ORF01 (1..528)	terminase small subunit	YP_009284651.1 putative terminase small subunit *Escherichia* phage vB_EcoS_NBD2	55/74 (170)	4e-60
ORF02 (530..2092)	terminase large subunit	AUV57173.1 terminase large subunit *Enterobacter phage Ec*_L1	81/91 (519)	0.0
ORF03 (2104..3471)	portal protein	AUV57174.1 portal protein *Enterobacter phage Ec*_L1	58/76 (426)	5e-179
ORF04 (3449..4210)	head morphogenesis protein	AUV57175.1 hypothetical protein Ec61 *Enterobacter phage Ec*_L1	52/70 (248)	2e-86
ORF05 (4220..5329)	scaffolding protein	YP_009280739.1 capsid and scaffold protein *Salmonella* phage phSE-2	58/73 (352)	1e-134
ORF06 (5382..5897)	capsid decoration protein	QEG10379.1 hypothetical protein KOX9_6 *Klebsiella* phage KOX9 (169)	64/73 (170)	9e-60
ORF07 (6017..6961)	major capsid protein	AUV57178.1 hypothetical protein Ec64 *Enterobacter phage Ec*_L1	78/88 (315)	0.0
ORF08 (7022..7363)	hypothetical protein	ATN93801.1 hypothetical protein *Escherichia* phage SRT8	43/54 (86)	2e-11
ORF09 (7407..7796)	putative head-tail adaptor	QEA09706.1 putative head-tail adaptor *Escherichia* phage Henu7	68/78 (128)	4e-59
ORF10 (7798..8169)	putative head completion protein	AUV57181.1 hypothetical protein Ec67 *Enterobacter phage Ec*_L1	67/78 (121)	2e-43
ORF11 (8162..8596)	putative neck protein	AUV57182.1 hypothetical protein Ec68 *Enterobacter phage Ec*_L1	69/83 (144)	9e-68
ORF12 (8596..9024)	putative tail completion protein	YP_007005387.1 hypothetical protein *Cronobacter* virus Esp2949-1	58/68 (138)	3e-47
ORF13 (9039..9695)	major tail protein	AUV57184.1 hypothetical protein Ec70 *Enterobacter phage Ec*_L1	68/82 (213)	5e-110
ORF14 (9780..10097)	tail assembly chaperone	QCW18522.1 tape measure chaperone *Escherichia* phage vB_EcoS_W011D (105)	52/69 (107)	3e-34
ORF15 (10160..10426)	hypothetical protein	YP_009280750.1 hypothetical protein *Salmonella* phage phSE-2	46/61 (83)	2e-14
ORF16 (10458..13472)	tape measure protein	YP_009280751.1 tail length tape-measure protein 1 *Salmonella* phage phSE-2	55/61 (1032)	0.0
ORF17 (13476..13823)	minor tail protein	YP_007005382.1 minor tail protein *Cronobacter* virus Esp2949-1	56/72 (113)	7e-42
ORF18 (13896..14651)	minor tail protein	AUV57189.1 minor tail protein *Enterobacter phage Ec*_L1	71/86 (251)	4e-139
ORF19 (14648..15382)	putative tail-associated protein	AUV57190.1 tail assembly protein *Enterobacter phage Ec*_L1	64/79 (248)	4e-117
ORF20 (15360..15959)	tail assembly protein	AUV57191.1 tail assembly protein *Enterobacter phage Ec*_L1	75/88 (199)	8e-109
ORF21 (16032..19568)	tail fiber protein	AUV57192.1 tail fiber protein *Enterobacter phage Ec*_L1	64/75 (1299)	0.0
ORF22 (19603..19902)	hypothetical protein	QGF21986.1 hypothetical protein *Erwinia* phage Midgardsormr38	39/58 (99)	1e-16
ORF23 (19906..20586)	structural protein	QGF21987.1 hypothetical protein *Erwinia* phage Midgardsormr38	45/61 (228)	4e-50

ORF24 (20671..22224)	tail fiber protein	YP_009618059.1 Stf, partial *Salmonella* virus SP126	41/58 (255)	8e-42
ORF25 complement (22299..22745)	single-stranded DNA-binding protein	AUV57196.1 hypothetical protein Ec82 *Enterobacter phage Ec*_L1	57/66 (160)	1e-53
ORF26 complement (22792..23478)	recombinase	AUV57197.1 recombinase *Enterobacter phage Ec*_L1	50/65 (229)	3e-71
ORF27 complement (23534..24580)	putative exodeoxyribonuclease VIII	AUV57198.1 exodeoxyribonuclease VIII *Enterobacter phage Ec*_L1	62/77 (346)	1e-155
ORF30 complement (25197..26147)	DNA primase/helicase	AWD92260.1 putative DNA primase/helicase Escherichia phage vB_EcoS_IME347	61/77 (309)	5e-138
ORF31 complement (26221..26628)	putative transcriptional regulator	QCW18508.1 putative transcriptional regulator *Escherichia* phage vB_EcoS_W011D	66/78 (132)	2e-58
ORF32 (26726..28759)	DNA helicase	AUV57115.1 DNA helicase *Enterobacter phage Ec*_L1	76/86 (676)	0.0
ORF33 (28743..29174)	putative nuclease containing VRR-NUC domain	SMH63966.1 VRR-NUC domain-containing protein *Escherichia* phage vB_Eco_swan01	66/75 (137)	3e-55
ORF34 (29238..29978)	DNA N-6-adenine-methyltransferase	SMH63965.1 Dam *Escherichia* phage vB_Eco_swan01	60/75 (244)	1e-104
ORF35 (29978..30211)	hypothetical protein	AVQ09765.1 hypothetical protein *Salmonella* phage vB_SenS_PHB07	43/61 (77)	2e-15
ORF36 (30255..30509)	hypothetical protein	AUV57119.1 hypothetical protein Ec05 *Enterobacter phage Ec*_L1	53/76 (77)	1e-22
ORF37 (30506..30754)	hypothetical protein	YP_009284690.1 hypothetical protein NBD2_66 *Escherichia* phage vB_EcoS_NBD2	36/55 (81)	1e-05
ORF38 (30800..31924)	putative phosphoesterase	YP_009226024.1 hypothetical protein KP36_062 *Klebsiella* phage KP36	70/83 (376)	0.0
ORF39 (31970..32497)	3'-phosphatase/5'-polynucleotide kinase	AUV57122.1 3'-phosphatase, 5'-polynucleotide kinase *Enterobacter phage Ec*_L1	74/84 (150)	8e-77
ORF40 (32494..33057)	putative deoxynucleoside monophosphate kinase	QEA09690.1 putative ATP-binding protein *Escherichia* phage Henu7	54/68 (177)	7e-61
ORF42 (33265..33435)	hypothetical protein	AWD92270.1 hypothetical protein Escherichia phage vB_EcoS_IME347	52/69 (56)	2e-11
ORF44 (33566..33784)	holin	AOZ65326.1 putative holin *Klebsiella* phage vB_KpnS_KpV522	73/85 (71)	1e-28
ORF45 (33786..34280)	endolysin	YP_009195400.1 endolysin *Klebsiella* phage KLPN1	65/81 (153)	1e-70
ORF46 (34277..34678)	spanin	YP_009284695.1 hypothetical protein NBD2_71 *Escherichia* phage vB_EcoS_NBD2	51/64 (124)	7e-28
ORF47 complement (34690..34893)	hypothetical protein	AUV57130.1 hypothetical protein Ec16 *Enterobacter phage Ec*_L1	72/88 (60)	7e-25
ORF48 complement (34871..35227)	hypothetical protein	YP_001285560.1 gp70 *Escherichia* virus TLS	57/76 (110)	1e-38
ORF49 complement	putative helicase	AUV57132.1 hypothetical protein Ec18 *Enterobacter phage Ec*_L1	72/87 (519)	0.0

(35230..36816)				
ORF50 complement (36885..37319)	hypothetical protein	YP_009226032.1 hypothetical protein KP36_070 *Klebsiella* phage KP36	58/73 (148)	3e-43
ORF52 complement (37523..38230)	DNA-cytosine methyltransferase	SMH63952.1 DNA-cytosine methylase *Escherichia* phage vB_Eco_swan01	61/70 (238)	5e-97
ORF53 complement (38240..38482)	hypothetical protein	AOZ65335.1 hypothetical protein kpv522_70 *Klebsiella* phage vB_KpnS_KpV522	48/60 (73)	5e-13
ORF56 complement (39249..39551)	hypothetical protein	AUV57140.1 hypothetical protein Ec26 *Enterobacter phage Ec*_L1	38/51 (85)	3e-06
ORF62 (40841..41137)	putative transcriptional regulator	WP_071882931.1 hypothetical protein *Pantoea* sp. PSNIH1	48/68 (79)	5e-14
ORF63 (41130..41351)	hypothetical protein	AWD92212.1 hypothetical protein Escherichia phage vB_EcoS_IME347	44/62 (66)	3e-09
ORF64 (41344..41631)	hypothetical protein	AFO12449.1 hypothetical protein *Salmonella* phage vB_SenS_AG11	57/75 (91)	3e-37
ORF66 (41854..42054)	hypothetical protein	QDH45820.1 hypothetical protein PSKM_gp63 *Pantoea* phage vB_PagM_PSKM	52/74 (66)	3e-17
ORF67 (42145..42378)	hypothetical protein	YP_006383655.1 hypothetical protein DIBBI_048 *Xanthomonas* phage vB_XveM_DIBBI	51/68 (75)	4e-18
ORF69 (42941..43183)	hypothetical protein	QMP82041.1 hypothetical protein KpV2811_075 *Klebsiella* virus KpV2811	29/35 (61)	1e-10
ORF70 (43146..43286)	hypothetical protein	QDH45735.1 hypothetical protein AAM37_gp64 *Pantoea* phage vB_PagM_AAM37	73/72 (51)	1e-32
ORF71 (43295..43501)	hypothetical protein	QDH45736.1 hypothetical protein AAM37_gp65 *Pantoea* phage vB_PagM_AAM37	78/80 (68)	2e-43
ORF73 (43753..44022)	DUF4884 domain-containing hypothetical protein	YP_398947.1 hypothetical protein rtp3 *Escherichia* virus Rtp	51/57 (68)	9e-12
ORF74 (44093..44392)	hypothetical protein	YP_004539086.1 hypothetical protein LIMEzero_ORF13 *Pantoea* phage LIMEzero	73/86 (51)	6e-19
ORF75 (44392..44847)	hypothetical protein	AZS06284.1 hypothetical protein MED16_gp44 *Pantoea* phage vB_PagS_MED16	49/60 (150)	4e-26
ORF77 (45292..45519)	hypothetical protein	AUV57153.1 hypothetical protein Ec39 *Enterobacter phage Ec*_L1	68/77 (75)	2e-31
ORF78 (45516..45701)	hypothetical protein	WP_010281918.1 hypothetical protein *Pectobacterium brasiliense*	50/74 (54)	1e-07
ORF83 complement (47194..48069)	hypothetical protein	AXF51433.1 hypothetical protein PAVTOK_5 *Erwinia* phage Pavtok	64/69 (59)	7e-21
ORF84 (48180..48779)	hypothetical protein	AUV57163.1 hypothetical protein Ec49 *Enterobacter phage Ec*_L1	32/47 (196)	7e-14
ORF87 (49919..50071)	hypothetical protein	AWD92225.1 hypothetical protein Escherichia phage vB_EcoS_IME347	49/65 (43)	7e-04
ORF89 (50191..50445)	hypothetical protein	ARM70402.1 hypothetical protein KOX1_78 *Klebsiella* phage KOX1	48/61 (84)	3e-19
ORF90 (50653..50838)	hypothetical protein	AUV57170.1 hypothetical protein Ec56 *Enterobacter phage Ec*_L1	44/71 (57)	1e-11

Table S4. Structural AAS23 proteins identified by MS.

Gene	Putative function	MW (KDa)	Peptide count	Sequence coverage (%)
ORF21	tail fiber protein	131.099	51	41.42
ORF16	tape measure protein	107.068	101	54.08
ORF24	tail fiber protein	53.933	28	54.54
ORF03	portal protein	51.229	23	57.14
ORF38	phosphoesterase	41.586	1	2.67
ORF05	scaffolding protein	39.652	4	11.11
ORF07	major capsid protein	34.627	26	61.46
ORF83	hypothetical protein	30.956	10	35.39
ORF04	portal protein	29.067	21	64.42
ORF18	minor tail protein	27.930	2	8.36
ORF23	structural protein	24.106	6	29.64
ORF13	neck protein	23.821	5	13.30
ORF20	tail assembly protein	20.769	5	27.63
ORF06	capsid decoration protein	17.393	7	28.65
ORF11	neck protein	16.059	3	21.52
ORF17	minor tail protein	12.771	1	7.82
ORF82	hypothetical protein	8.835	3	38.27

AAS23 specific ORF with no reliable identity to database entries is underlined.

Table S5. Top matches for BLAST-based alignments of whole genome sequences of AAS23 and its closest relatives generated using PASC.

The overall nucleotide sequence identity (%)	Reference Sequence	Bacteriophage	Genus within the family *Drexlerviridae*
46.58%	NC_042122.1	*Enterobacter* phage Ec_L1	*Eclunavirus*
42.37%	NC_031050.1	*Esherichia* phage vB_EcoS_NBD2	*Vilniusvirus*
41.84%	MN019128.1	*Escherichia* phage Henu7	unclassified *Drexlerviridae*
41.56%	NC_047960.1	*Escherichia* phage vB_EcoS_IME347	*Badaguanvirus*
41.49%	MK778457.1	*Escherichia* phage vB_EcoS_W011D	unclassified *Drexlerviridae*
40.83%	NC_047825.1	*Klebsiella* phage KOX1	*Webervirus*
40.56%	NC_042043.1	*Escherichia* phage SRT8	*Sertoctavirus*
40.50%	NC_019509.1	*Cronobacter* phage ESP2949-1	*Tunavirinae*
40.41%	NC_047784.1	*Klebsiella* phage vB_KpnS_KpV522	*Webervirus*
40.08%	LT841304.1	*Escherichia* phage vB_EcoS_swan01	*Warwickvirus*

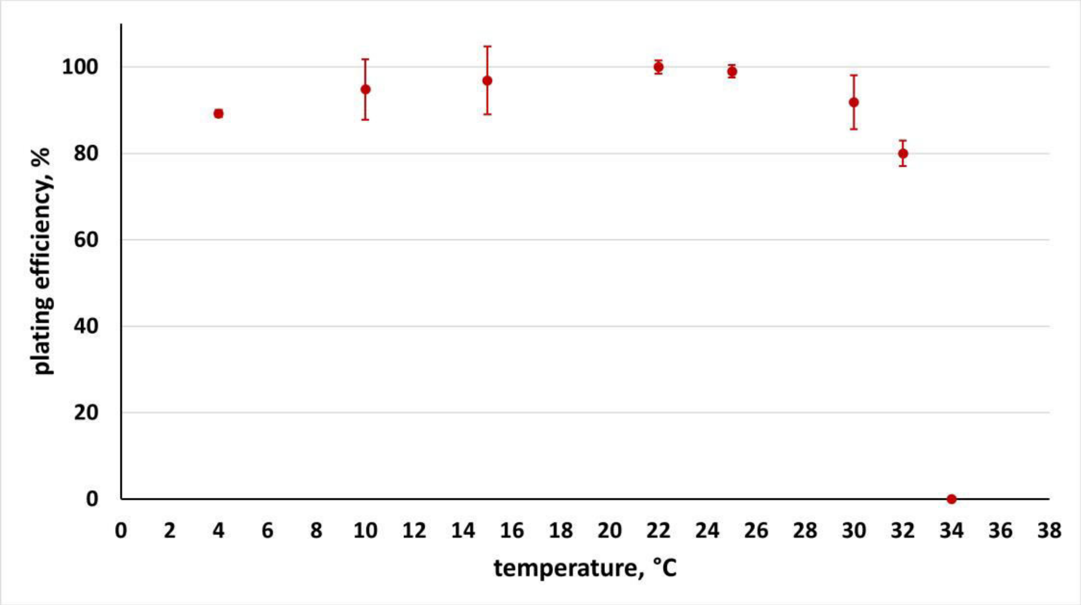

Figure S1. Effect of temperature on the plating efficiency of phage AAS23. Each point represents the mean of three independent experiments. Error bars indicate standard deviation.

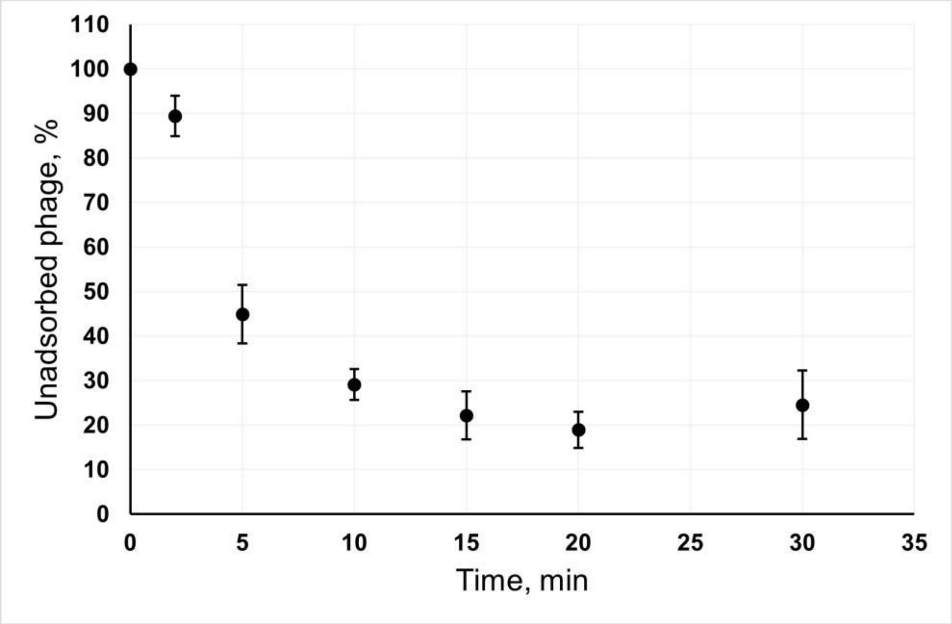

Figure S2. Adsorption rate of phage AAS23. Each point represents the mean of three independent experiments. Error bars indicate standard deviation.

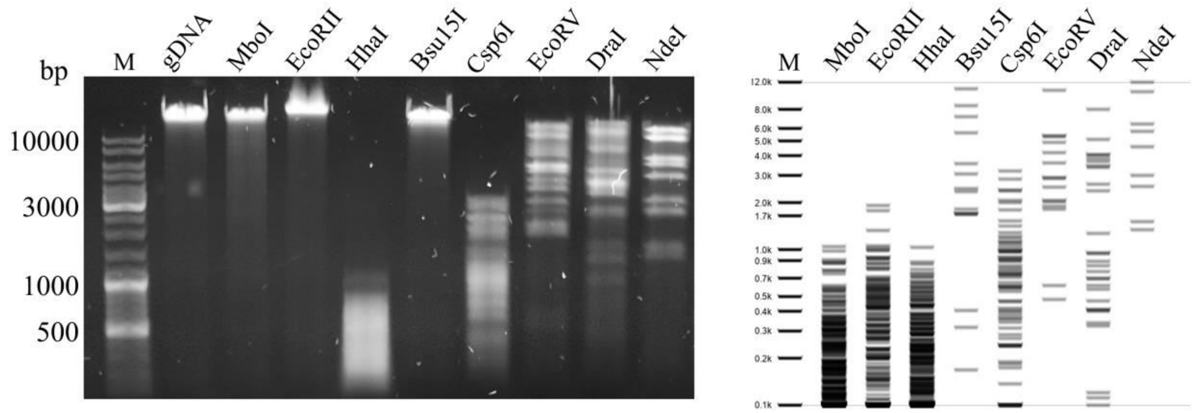

Figure S3. Restriction digestion patterns of AAS23 genomic DNA. M– GeneRuler™ DNA Ladder Mix (Thermo Fisher Scientific); gDNA – undigested genomic DNA of AAS23. Methylation sensitivity: MboI (dam methylation-sensitive); EcoRII (dcm methylation-sensitive); HhaI (CpG methylation-sensitive); Bsu15I (CpG methylation-sensitive, dam methylation-sensitive); Csp6I, EcoRV, DraI, NdeI (not dam methylation-sensitive, not dcm methylation-sensitive, not CpG methylation-sensitive). On the right side of the gel is the representation of the expected restriction pattern.

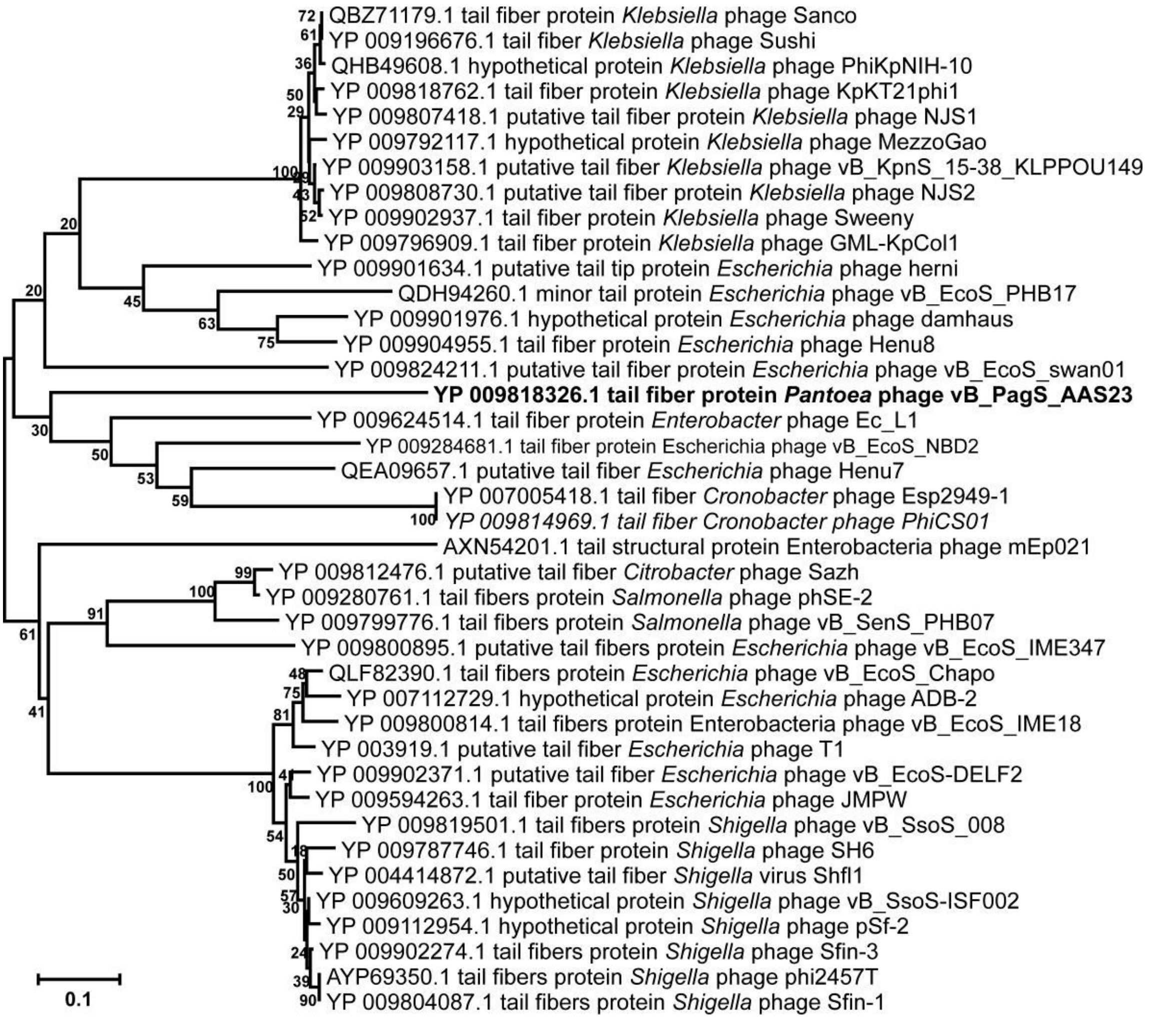

Figure S4. Neighbor-joining tree analysis based on the alignment of the amino acid sequences of AAS23 tail fiber protein (gp24) and its closest homologues. The percentage of replicate trees in which the associated taxa clustered together in the bootstrap test is shown next to the branches.

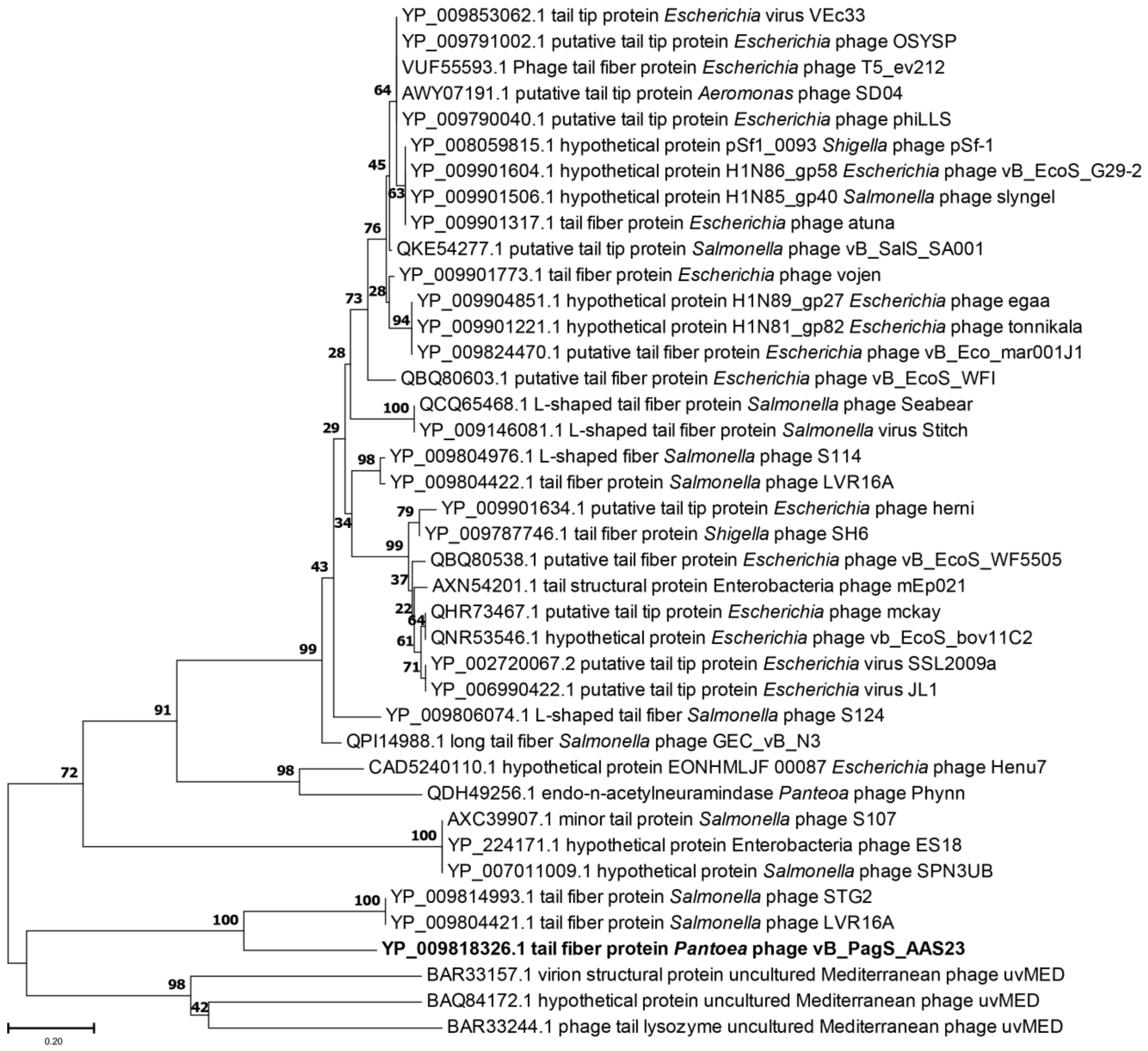

Figure S5. Neighbor-joining tree analysis based on the alignment of the amino acid sequences of conserved Peptidase_S74 domain (aa 402 to 459) of AAS23 tail fiber protein (gp24) and its closest homologues. The percentage of replicate trees in which the associated taxa clustered together in the bootstrap test is shown next to the branches.
