# Supplementary material for: Pantoea Bacteriophage vB_PagS_AAS23: A Singleton of the Genus Sauletekiovirus
Source: Microorganisms. 2021 Mar 23;9(3):668. doi: 10.3390/microorganisms9030668 (PMC8004638; doi:10.3390/microorganisms9030668)
Supplement: Supplementary file 1 [file microorganisms-09-00668-s001.zip › Supplementary material/Microoganisms Suppl.doc]

**Table S1.** **A list of *Pantoea* bacteriophages with completely sequenced genomes, which have been published and/or deposited in the public databases.**

| Phage | GenBank accession no. | Family | Genome size (bp) | Reference |
| --- | --- | --- | --- | --- |
| Phynn | MN038175.1 | *Myoviridae* | 173720 | unpublished |
| vB_PagM_LIET2 | [MK388689.1](https://www.ncbi.nlm.nih.gov/nuccore/MK388689.1) | *Myoviridae* | 74710 | unpublished |
| Kyle | NC048796.1 | *Myoviridae* | 73168 | unpublished |
| vB_PagM_SSEM1 | [MT230534.1](https://www.ncbi.nlm.nih.gov/nuccore/MT230534.1) | *Myoviridae* | 54982 | unpublished |
| vB_PagM_AAM37 | [MK798143.1](https://www.ncbi.nlm.nih.gov/nuccore/MK798143.1) | *Myoviridae* | 49990 | unpublished |
| vB_PagM_PSKM | [MK798144.1](https://www.ncbi.nlm.nih.gov/nuccore/MK798144.1) | *Myoviridae* | 49935 | unpublished |
| vB_PagM_AAM22 | [MK798142.1](https://www.ncbi.nlm.nih.gov/nuccore/MK798142.1) | *Myoviridae* | 49744 | [11] |
| vB_PagS_AAS21 | [MK770119.1](https://www.ncbi.nlm.nih.gov/nuccore/MK770119.1) | *Siphoviridae* | 116649 | [12] |
| vB_PagS_Vid5 | [MG948468.1](https://www.ncbi.nlm.nih.gov/nuccore/MG948468.1) | *Siphoviridae* | 61437 | [13] |
| **vB_PagS_AAS23** | [**MK095606.1**](https://www.ncbi.nlm.nih.gov/nuccore/MK095606.1) | ***Siphoviridae*** | **51170** | **[this study]** |
| vB_PagS_MED16 | [MK095605.1](https://www.ncbi.nlm.nih.gov/nuccore/MK095605.1) | *Siphoviridae* | 46103 | unpublished |
| LIMElight | [FR687252.1](https://www.ncbi.nlm.nih.gov/nuccore/FR687252.1) | *Podoviridae* | 44546 | [14] |
| LIMEzero | [FR751545.1](https://www.ncbi.nlm.nih.gov/nuccore/FR751545.1) | *Podoviridae* | 43032 | [14] |
| vB_PagP-SK1 | [MN450150.1](https://www.ncbi.nlm.nih.gov/nuccore/MN450150.1) | *Podoviridae* | 39938 | [15] |

**Table S2.** Bacterial strains used in this study to determine the host range of phage AAS23.

| Strain | Relevant characteristics | Source or reference |
| --- | --- | --- |
| *Acinetobacter baumannii* #46 |  | Prof. E. ų |
| *Citrobacter freundii* |  | Prof. E. Sužiedėlienė |
| *Erwinia carotovora* 8982 |  | Prof. E. Sužiedėlienė |
| *Erwinia carotovora* 961‒63 |  | Prof. E. Sužiedėlienė |
| *Erwinia* *piriflorinigrans* 26166 | type strain | DSMZ |
| *Erwinia billingiae* 17872 | type strain | DSMZ |
| *Escherichia coli* B40 | *sup*D | Dr. L. W. Black |
| *Escherichia coli* B^E^ | *sup*^0^ | Dr. L. W. Black |
| *Escherichia coli* BL21 | F^–^ *dcm ompT hsdS* (rB^–^mB^–^) *gal* | Novagen |
| *Escherichia coli* BW25113 | [Δ(araD–araB)567 Δ(rhaD–rhaB)568 ΔlacZ4787 (::rrnB-3) hsdR514 rph-1] | [27] |
| *Escherichia coli* DH10B | F^–^ *endA1 recA1 galE15 galK16 nupG rpsL* Δ*lacX74* Φ80*lacZ*ΔM15 *araD139* Δ(*ara,leu*)7697 *mcrA* Δ(*mrr*-*hsdRMS*-*mcrBC*) λ^-^ | Invitrogen |
| *Escherichia coli* MG1655 | F^-^ lambda^-^ *ilvG*^-^ *rfb*-50 *rph*-1 | Prof. E. Sužiedėlienė |
| *Escherichia coli* MH1 | *araD139* Δ*lacX74 galU galK hsr hsm rpsL* | Dr. K. N. Kreuzer |
| *Klebsiella* sp. KV-3 | Veterinary isolate, Amp^r^, Str^r^, Tet^r^, Kan^s^, Gm^s^, Nc^s^,Cl^r/s^ | [28] |
| *Pantoea agglomerans* ARC | environmental isolate | [13] |
| *Pantoea agglomerans* AUR^a^ | environmental isolate | [13] |
| *Pantoea agglomerans* BSL | environmental isolate | [13] |
| *Pantoea agglomerans* DDM | environmental isolate | [13] |
| *Pantoea agglomerans* MMG | environmental isolate | [13] |
| *Pantoea agglomerans* SER | environmental isolate | [13] |
| *Pantoea agglomerans* 3493 | type strain | DSMZ |
| *Pantoea conspicua* 24241 | type strain | DSMZ |
| *Pseudomonas aeruginosa* PAO1 |  | Prof. E. Sužiedėlienė |
| *Salmonella enterica* ser. Typhimurium 292 |  | Prof. E. Sužiedėlienė |

a – AAS23-sensitive strain.

**Table S3.** **AAS23 ORFs-having homologues with reliable identity (E-values >0.001) in other viruses or cellular organisms.**

| AAS23 ORF  (position) | Predicted function | Significant match | Identity aa %/  similarity aa% (length of the overlapping segment) | | E value |
| --- | --- | --- | --- | --- | --- |
| ORF01  (1..528) | terminase small subunit | [YP_009284651.1](https://www.ncbi.nlm.nih.gov/protein/YP_009284651?report=genbank&log$=protalign&blast_rank=1&RID=GJBFDT6J014) putative terminase small subunit  *Escherichia* phage vB_EcoS_NBD2 | | 55/74 (170) | 4e-60 |
| ORF02  (530..2092) | terminase large subunit | [AUV57173.1](https://www.ncbi.nlm.nih.gov/protein/AUV57173?report=genbank&log$=protalign&blast_rank=1&RID=GJBKRZ2P015) terminase large subunit  *Enterobacter phage Ec*_L1 | | 81/91 (519) | 0.0 |
| ORF03  (2104..3471) | portal protein | [AUV57174.1](https://www.ncbi.nlm.nih.gov/protein/AUV57174?report=genbank&log$=protalign&blast_rank=1&RID=GJCXTSXS01R) portal protein  *Enterobacter phage Ec*_L1 | | 58/76 (426) | 5e-179 |
| ORF04  (3449..4210) | head morphogenesis protein | [AUV57175.1](https://www.ncbi.nlm.nih.gov/protein/AUV57175?report=genbank&log$=protalign&blast_rank=1&RID=GJD33B4101R) hypothetical protein Ec61  *Enterobacter phage Ec*_L1 | | 52/70 (248) | 2e-86 |
| ORF05  (4220..5329) | scaffolding protein | [YP_009280739.1](https://www.ncbi.nlm.nih.gov/protein/YP_009280739?report=genbank&log$=protalign&blast_rank=1&RID=GJDDCUXS014) capsid and scaffold protein  *Salmonella* phage phSE-2 | | 58/73 (352) | 1e-134 |
| ORF06  (5382..5897) | capsid decoration protein | [QEG10379.1](https://www.ncbi.nlm.nih.gov/protein/QEG10379.1?report=genbank&log$=prottop&blast_rank=2&RID=3Y7KTT7X016) hypothetical protein KOX9_6  *Klebsiella* phage KOX9 (169) | | 64/73 (170) | 9e-60 |
| ORF07  (6017..6961) | major capsid protein | [AUV57178.1](https://www.ncbi.nlm.nih.gov/protein/AUV57178?report=genbank&log$=protalign&blast_rank=1&RID=GJDWFUWF014) hypothetical protein Ec64  *Enterobacter phage Ec*_L1 | | 78/88 (315) | 0.0 |
| ORF08  (7022..7363) | hypothetical protein | [ATN93801.1](https://www.ncbi.nlm.nih.gov/protein/ATN93801?report=genbank&log$=protalign&blast_rank=1&RID=GJDYAXES014) hypothetical protein  *Escherichia* phage SRT8 | | 43/54 (86) | 2e-11 |
| ORF09  (7407..7796) | putative head-tail adaptor | [QEA09706.1](https://www.ncbi.nlm.nih.gov/protein/QEA09706.1?report=genbank&log$=prottop&blast_rank=2&RID=3Y7XE3Y7014) putative head-tail adaptor  *Escherichia* phage Henu7 | | 68/78 (128) | 4e-59 |
| ORF10  (7798..8169) | putative head completion protein | [AUV57181.1](https://www.ncbi.nlm.nih.gov/protein/AUV57181?report=genbank&log$=protalign&blast_rank=1&RID=GJE8R94F014) hypothetical protein Ec67  *Enterobacter phage Ec*_L1 | | 67/78 (121) | 2e-43 |
| ORF11  (8162..8596) | putative neck protein | [AUV57182.1](https://www.ncbi.nlm.nih.gov/protein/AUV57182?report=genbank&log$=protalign&blast_rank=1&RID=GJENW1SC014) hypothetical protein Ec68  *Enterobacter phage Ec*_L1 | | 69/83 (144) | 9e-68 |
| ORF12  (8596..9024) | putative tail completion protein | [YP_007005387.1](https://www.ncbi.nlm.nih.gov/protein/YP_007005387?report=genbank&log$=protalign&blast_rank=1&RID=GJERBK82014) hypothetical protein  *Cronobacter* virus Esp2949-1 | | 58/68 (138) | 3e-47 |
| ORF13  (9039..9695) | major tail protein | [AUV57184.1](https://www.ncbi.nlm.nih.gov/protein/AUV57184?report=genbank&log$=protalign&blast_rank=1&RID=GJF5VTUP014) hypothetical protein Ec70  *Enterobacter phage Ec*_L1 | | 68/82 (213) | 5e-110 |
| ORF14  (9780..10097) | tail assembly chaperone | [QCW18522.1](https://www.ncbi.nlm.nih.gov/protein/QCW18522.1?report=genbank&log$=prottop&blast_rank=2&RID=3Y8KBUPD016) tape measure chaperone  *Escherichia* phage vB_EcoS_W011D (105) | | 52/69 (107) | 3e-34 |
| ORF15  (10160..10426) | hypothetical protein | [YP_009280750.1](https://www.ncbi.nlm.nih.gov/protein/YP_009280750?report=genbank&log$=protalign&blast_rank=1&RID=GJFP3M34014) hypothetical protein  *Salmonella* phage phSE-2 | | 46/61 (83) | 2e-14 |
| ORF16  (10458..13472) | tape measure protein | YP_009280751.1 tail length tape-measure protein 1  *Salmonella* phage phSE-2 | | 55/61 (1032) | 0.0 |
| ORF17  (13476..13823) | minor tail protein | [YP_007005382.1](https://www.ncbi.nlm.nih.gov/protein/YP_007005382?report=genbank&log$=protalign&blast_rank=1&RID=GJM3NWWP015) minor tail protein  *Cronobacter* virus Esp2949-1 | | 56/72 (113) | 7e-42 \| |
| ORF18  (13896..14651) | minor tail protein | [AUV57189.1](https://www.ncbi.nlm.nih.gov/protein/AUV57189?report=genbank&log$=protalign&blast_rank=1&RID=GJM5B8K3015) minor tail protein  *Enterobacter phage Ec*_L1 | | 71/86 (251) | 4e-139 |
| ORF19  (14648..15382) | putative tail-associated protein | [AUV57190.1](https://www.ncbi.nlm.nih.gov/protein/AUV57190?report=genbank&log$=protalign&blast_rank=1&RID=GJMG89Y101R) tail assembly protein  *Enterobacter phage Ec*_L1 | | 64/79 (248) | 4e-117 |
| ORF20  (15360..15959) | tail assembly protein | [AUV57191.1](https://www.ncbi.nlm.nih.gov/protein/AUV57191?report=genbank&log$=protalign&blast_rank=1&RID=GJMHXE6G01R) tail assembly protein  *Enterobacter phage Ec*_L1 | | 75/88 (199) | 8e-109 |
| ORF21  (16032..19568) | tail fiber protein | [AUV57192.1](https://www.ncbi.nlm.nih.gov/protein/AUV57192?report=genbank&log$=protalign&blast_rank=1&RID=GJN06ZKS014) tail fiber protein  *Enterobacter phage Ec*_L1 | | 64/75 (1299) | 0.0 |
| ORF22  (19603..19902) | hypothetical protein | [QGF21986.1](https://www.ncbi.nlm.nih.gov/protein/QGF21986.1?report=genbank&log$=prottop&blast_rank=2&RID=3Y9JNA9Z016) hypothetical protein  *Erwinia* phage Midgardsormr38 | | 39/58 (99) | 1e-16 |
| ORF23  (19906..20586) | structural protein | [QGF21987.1](https://www.ncbi.nlm.nih.gov/protein/QGF21987.1?report=genbank&log$=prottop&blast_rank=2&RID=3Y9K8BMN016) hypothetical protein  *Erwinia* phage Midgardsormr38 | | 45/61 (228) | 4e-50 |
| ORF24  (20671..22224) | tail fiber protein | [YP_009618059.1](https://www.ncbi.nlm.nih.gov/protein/YP_009618059.1?report=genbank&log$=prottop&blast_rank=2&RID=3Y9M3N6N016) Stf, partial  *Salmonella* virus SP126 | | 41/58 (255) | 8e-42 |
| ORF25  complement (22299..22745) | single-stranded DNA-binding protein | [AUV57196.1](https://www.ncbi.nlm.nih.gov/protein/AUV57196?report=genbank&log$=protalign&blast_rank=1&RID=GJPTKBA501R) hypothetical protein Ec82  *Enterobacter phage Ec*_L1 | | 57/66 (160) | 1e-53 |
| ORF26  complement (22792..23478) | recombinase | [AUV57197.1](https://www.ncbi.nlm.nih.gov/protein/AUV57197?report=genbank&log$=protalign&blast_rank=1&RID=GJPV1N5701R) recombinase  *Enterobacter phage Ec*_L1 | | 50/65 (229) | 3e-71 |
| ORF27  complement (23534..24580) | putative exodeoxyribonuclease VIII | [AUV57198.1](https://www.ncbi.nlm.nih.gov/protein/AUV57198?report=genbank&log$=protalign&blast_rank=1&RID=GJRAN66D01R) exodeoxyribonuclease VIII  *Enterobacter phage Ec*_L1 | | 62/77 (346) | 1e-155 |
| ORF30  complement (25197..26147) | DNA primase/helicase | [AWD92260.1](https://www.ncbi.nlm.nih.gov/protein/AWD92260?report=genbank&log$=protalign&blast_rank=1&RID=GJRSMH9F01R) putative DNA primase/helicase  Escherichia phage vB_EcoS_IME347 | | 61/77 (309) | 5e-138 |
| ORF31  complement (26221..26628) | putative transcriptional regulator | [QCW18508.1](https://www.ncbi.nlm.nih.gov/protein/QCW18508.1?report=genbank&log$=prottop&blast_rank=2&RID=3YAKAGXN016) putative transcriptional regulator  *Escherichia* phage vB_EcoS_W011D | | 66/78 (132) | 2e-58 |
| ORF32  (26726..28759) | DNA helicase | [AUV57115.1](https://www.ncbi.nlm.nih.gov/protein/AUV57115?report=genbank&log$=protalign&blast_rank=1&RID=GJSC9V18015) DNA helicase  *Enterobacter phage Ec*_L1 | | 76/86 (676) | 0.0 |
| ORF33  (28743..29174) | putative nuclease containing VRR-NUC domain | [SMH63966.1](https://www.ncbi.nlm.nih.gov/protein/SMH63966?report=genbank&log$=protalign&blast_rank=1&RID=GJSKD3AZ015) VRR-NUC domain-containing protein  *Escherichia* phage vB_Eco_swan01 | | 66/75 (137) | 3e-55 |
| ORF34  (29238..29978) | DNA N-6-adenine-methyltransferase | [SMH63965.1](https://www.ncbi.nlm.nih.gov/protein/SMH63965?report=genbank&log$=protalign&blast_rank=1&RID=GJSN4132014) Dam  *Escherichia* phage vB_Eco_swan01 | | 60/75 (244) | 1e-104 |
| ORF35  (29978..30211) | hypothetical protein | [AVQ09765.1](https://www.ncbi.nlm.nih.gov/protein/AVQ09765?report=genbank&log$=protalign&blast_rank=1&RID=GJT209W201R) hypothetical protein  *Salmonella* phage vB_SenS_PHB07 | | 43/61 (77) | 2e-15 |
| ORF36  (30255..30509) | hypothetical protein | [AUV57119.1](https://www.ncbi.nlm.nih.gov/protein/AUV57119?report=genbank&log$=protalign&blast_rank=1&RID=GJT3AD0V01R) hypothetical protein Ec05  *Enterobacter phage Ec*_L1 | | 53/76 (77) | 1e-22 |
| ORF37  (30506..30754) | hypothetical protein | [YP_009284690.1](https://www.ncbi.nlm.nih.gov/protein/YP_009284690?report=genbank&log$=protalign&blast_rank=1&RID=GJTB1B7601R) hypothetical protein NBD2_66  *Escherichia* phage vB_EcoS_NBD2 | | 36/55 (81) | 1e-05 |
| ORF38  (30800..31924) | putative phosphoesterase | [YP_009226024.1](https://www.ncbi.nlm.nih.gov/protein/YP_009226024?report=genbank&log$=protalign&blast_rank=1&RID=GJTCW26N01R) hypothetical protein KP36_062  *Klebsiella* phage KP36 | | 70/83 (376) | 0.0 |
| ORF39  (31970..32497) | 3'-phosphatase/5'-polynucleotide kinase | [AUV57122.1](https://www.ncbi.nlm.nih.gov/protein/AUV57122?report=genbank&log$=protalign&blast_rank=1&RID=GJTT3SDM015) 3'-phosphatase, 5'-polynucleotide kinase  *Enterobacter phage Ec*_L1 | | 74/84 (150) | 8e-77 |
| ORF40  (32494..33057) | putative deoxynucleoside monophosphate kinase | [QEA09690.1](https://www.ncbi.nlm.nih.gov/protein/QEA09690.1?report=genbank&log$=prottop&blast_rank=2&RID=3YB5GPH5016) putative ATP-binding protein  *Escherichia* phage Henu7 | | 54/68 (177) | 7e-61 |
| ORF42  (33265..33435) | hypothetical protein | [AWD92270.1](https://www.ncbi.nlm.nih.gov/protein/AWD92270?report=genbank&log$=protalign&blast_rank=1&RID=GJUV541K01R) hypothetical protein  Escherichia phage vB_EcoS_IME347 | | 52/69 (56) | 2e-11 |
| ORF44  (33566..33784) | holin | [AOZ65326.1](https://www.ncbi.nlm.nih.gov/protein/AOZ65326?report=genbank&log$=protalign&blast_rank=1&RID=GJVCP4ZK01R) putative holin  *Klebsiella* phage vB_KpnS_KpV522 | | 73/85 (71) | 1e-28 |
| ORF45  (33786..34280) | endolysin | [YP_009195400.1](https://www.ncbi.nlm.nih.gov/protein/YP_009195400?report=genbank&log$=protalign&blast_rank=1&RID=GJVJSHYS01R) endolysin  *Klebsiella* phage KLPN1 | | 65/81 (153) | 1e-70 |
| ORF46  (34277..34678) | spanin | [YP_009284695.1](https://www.ncbi.nlm.nih.gov/protein/YP_009284695?report=genbank&log$=protalign&blast_rank=1&RID=GJVMHHG201R) hypothetical protein NBD2_71  *Escherichia* phage vB_EcoS_NBD2 | | 51/64 (124) | 7e-28 |
| ORF47  complement (34690..34893) | hypothetical protein | [AUV57130.1](https://www.ncbi.nlm.nih.gov/protein/AUV57130?report=genbank&log$=protalign&blast_rank=1&RID=GJWDDK7Y015) hypothetical protein Ec16  *Enterobacter phage Ec*_L1 | | 72/88 (60) | 7e-25 |
| ORF48  complement (34871..35227) | hypothetical protein | [YP_001285560.1](https://www.ncbi.nlm.nih.gov/protein/YP_001285560?report=genbank&log$=protalign&blast_rank=1&RID=GJWR491C015) gp70  *Escherichia* virus TLS | | 57/76 (110) | 1e-38 |
| ORF49  complement (35230..36816) | putative helicase | [AUV57132.1](https://www.ncbi.nlm.nih.gov/protein/AUV57132?report=genbank&log$=protalign&blast_rank=1&RID=GJX9AVUN015) hypothetical protein Ec18  *Enterobacter phage Ec*_L1 | | 72/87 (519) | 0.0 |
| ORF50  complement (36885..37319) | hypothetical protein | [YP_009226032.1](https://www.ncbi.nlm.nih.gov/protein/YP_009226032?report=genbank&log$=protalign&blast_rank=1&RID=GJXC22UE014) hypothetical protein KP36_070  *Klebsiella* phage KP36 | | 58/73 (148) | 3e-43 |
| ORF52  complement (37523..38230) | DNA-cytosine methyltransferase | [SMH63952.1](https://www.ncbi.nlm.nih.gov/protein/SMH63952?report=genbank&log$=protalign&blast_rank=1&RID=GJY3Z77W01R) DNA-cytosine methylase  *Escherichia* phage vB_Eco_swan01 | | 61/70 (238) | 5e-97 |
| ORF53  complement (38240..38482) | hypothetical protein | [AOZ65335.1](https://www.ncbi.nlm.nih.gov/protein/AOZ65335?report=genbank&log$=protalign&blast_rank=1&RID=GK1FRSJ2014) hypothetical protein kpv522_70  *Klebsiella* phage vB_KpnS_KpV522 | | 48/60 (73) | 5e-13 |
| ORF56  complement (39249..39551) | hypothetical protein | [AUV57140.1](https://www.ncbi.nlm.nih.gov/protein/AUV57140?report=genbank&log$=protalign&blast_rank=1&RID=GK310MYR014) hypothetical protein Ec26  *Enterobacter phage Ec*_L1 | | 38/51 (85) | 3e-06 |
| ORF62  (40841..41137) | putative transcriptional regulator | [WP_071882931.1](https://www.ncbi.nlm.nih.gov/protein/WP_071882931?report=genbank&log$=protalign&blast_rank=1&RID=GK2UTU7V014) hypothetical protein  *Pantoea* sp. PSNIH1 | | 48/68 (79) | 5e-14 |
| ORF63  (41130..41351) | hypothetical protein | [AWD92212.1](https://www.ncbi.nlm.nih.gov/protein/AWD92212?report=genbank&log$=protalign&blast_rank=1&RID=GK358E3C015) hypothetical protein  Escherichia phage vB_EcoS_IME347 | | 44/62 (66) | 3e-09 |
| ORF64  (41344..41631) | hypothetical protein | [AFO12449.1](https://www.ncbi.nlm.nih.gov/protein/AFO12449?report=genbank&log$=protalign&blast_rank=1&RID=GK3DEY2V015) hypothetical protein  *Salmonella* phage vB_SenS_AG11 | | 57/75 (91) | 3e-37 |
| ORF66  (41854..42054) | hypothetical protein | [QDH45820.1](https://www.ncbi.nlm.nih.gov/protein/QDH45820.1?report=genbank&log$=prottop&blast_rank=2&RID=3YCF2H8J014) hypothetical protein PSKM_gp63  *Pantoea* phage vB_PagM_PSKM | | 52/74 (66) | 3e-17 |
| ORF67  (42145..42378) | hypothetical protein | [YP_006383655.1](https://www.ncbi.nlm.nih.gov/protein/YP_006383655?report=genbank&log$=protalign&blast_rank=1&RID=GK3WNWTD014) hypothetical protein DIBBI_048  *Xanthomonas* phage vB_XveM_DIBBI | | 51/68 (75) | 4e-18 |
| ORF69  (42941..43183) | hypothetical protein | [QMP82041.1](https://www.ncbi.nlm.nih.gov/protein/QMP82041.1?report=genbank&log$=prottop&blast_rank=2&RID=1JMEMZP4016) hypothetical protein KpV2811_075  *Klebsiella* virus KpV2811 | | 29/35 (61) | 1e-10 |
| ORF70  (43146..43286) | hypothetical protein | [QDH45735.1](https://www.ncbi.nlm.nih.gov/protein/QDH45735.1?report=genbank&log$=prottop&blast_rank=2&RID=3YCX0P79016) hypothetical protein AAM37_gp64  *Pantoea* phage vB_PagM_AAM37 | | 73/72 (51) | 1e-32 |
| ORF71  (43295..43501) | hypothetical protein | [QDH45736.1](https://www.ncbi.nlm.nih.gov/protein/QDH45736.1?report=genbank&log$=prottop&blast_rank=2&RID=3YCXVYJJ014) hypothetical protein AAM37_gp65  *Pantoea* phage vB_PagM_AAM37 | | 78/80 (68) | 2e-43 |
| ORF73  (43753..44022) | DUF4884 domain-containing hypothetical protein | [YP_398947.1](https://www.ncbi.nlm.nih.gov/protein/YP_398947?report=genbank&log$=protalign&blast_rank=2&RID=GK58XJNA015) hypothetical protein rtp3  *Escherichia* virus Rtp | | 51/57 (68) | 9e-12 |
| ORF74  (44093..44392) | hypothetical protein | [YP_004539086.1](https://www.ncbi.nlm.nih.gov/protein/YP_004539086?report=genbank&log$=protalign&blast_rank=1&RID=GK5GVAG6015) hypothetical protein LIMEzero_ORF13  *Pantoea* phage LIMEzero | | 73/86 (51) | 6e-19 |
| ORF75  (44392..44847) | hypothetical protein | [AZS06284.1](https://www.ncbi.nlm.nih.gov/protein/AZS06284.1?report=genbank&log$=prottop&blast_rank=2&RID=3YD7RC6F016) hypothetical protein MED16_gp44  *Pantoea* phage vB_PagS_MED16 | | 49/60 (150) | 4e-26 |
| ORF77  (45292..45519) | hypothetical protein | [AUV57153.1](https://www.ncbi.nlm.nih.gov/protein/AUV57153?report=genbank&log$=protalign&blast_rank=1&RID=GK666448014) hypothetical protein Ec39  *Enterobacter phage Ec*_L1 | | 68/77 (75) | 2e-31 |
| ORF78  (45516..45701) | hypothetical protein | [WP_010281918.1](https://www.ncbi.nlm.nih.gov/protein/WP_010281918.1?report=genbank&log$=prottop&blast_rank=2&RID=3YDNGEGX014) hypothetical protein  *Pectobacterium brasiliense* | | 50/74 (54) | 1e-07 |
| ORF83  complement (47194..48069) | hypothetical protein | [AXF51433.1](https://www.ncbi.nlm.nih.gov/protein/AXF51433.1?report=genbank&log$=prottop&blast_rank=2&RID=3YE1HMZM014) hypothetical protein PAVTOK_5  *Erwinia* phage Pavtok | | 64/69 (59) | 7e-21 |
| ORF84  (48180..48779) | hypothetical protein | [AUV57163.1](https://www.ncbi.nlm.nih.gov/protein/AUV57163?report=genbank&log$=protalign&blast_rank=1&RID=GK8SFP1T015) hypothetical protein Ec49  *Enterobacter phage Ec*_L1 | | 32/47 (196) | 7e-14 |
| ORF87  (49919..50071) | hypothetical protein | [AWD92225.1](https://www.ncbi.nlm.nih.gov/protein/AWD92225?report=genbank&log$=protalign&blast_rank=1&RID=GK99KT1H014) hypothetical protein  Escherichia phage vB_EcoS_IME347 | | 49/65 (43) | 7e-04 |
| ORF89  (50191..50445) | hypothetical protein | [ARM70402.1](https://www.ncbi.nlm.nih.gov/protein/ARM70402?report=genbank&log$=protalign&blast_rank=1&RID=GK9F8NCB014) hypothetical protein KOX1_78  *Klebsiella* phage KOX1 | | 48/61 (84) | 3e-19 |
| ORF90  (50653..50838) | hypothetical protein | [AUV57170.1](https://www.ncbi.nlm.nih.gov/protein/AUV57170?report=genbank&log$=protalign&blast_rank=1&RID=GK9NGTFW014) hypothetical protein Ec56  *Enterobacter phage Ec*_L1 | | 44/71 (57) | 1e-11 |

**Table S4.** Structural AAS23 proteins identified by MS.

| Gene | Putative function | MW (KDa) | Peptide count | Sequence coverage (%) |
| --- | --- | --- | --- | --- |
| ORF21 | tail fiber protein | 131.099 | 51 | 41.42 |
| ORF16 | tape measure protein | 107.068 | 101 | 54.08 |
| ORF24 | tail fiber protein | 53.933 | 28 | 54.54 |
| ORF03 | portal protein | 51.229 | 23 | 57.14 |
| ORF38 | phosphoesterase | 41.586 | 1 | 2.67 |
| ORF05 | scaffolding protein | 39.652 | 4 | 11.11 |
| ORF07 | major capsid protein | 34.627 | 26 | 61.46 |
| ORF83 | hypothetical protein | 30.956 | 10 | 35.39 |
| ORF04 | portal protein | 29.067 | 21 | 64.42 |
| ORF18 | minor tail protein | 27.930 | 2 | 8.36 |
| ORF23 | structural protein | 24.106 | 6 | 29.64 |
| ORF13 | neck protein | 23.821 | 5 | 13.30 |
| ORF20 | tail assembly protein | 20.769 | 5 | 27.63 |
| ORF06 | capsid decoration protein | 17.393 | 7 | 28.65 |
| ORF11 | neck protein | 16.059 | 3 | 21.52 |
| ORF17 | minor tail protein | 12.771 | 1 | 7.82 |
| ORF82 | hypothetical protein | 8.835 | 3 | 38.27 |

AAS23 specific ORF with no reliable identity to database entries is underlined.

**Table S5**. Top matches for BLAST-based alignments of whole genome sequences of AAS23 and its closest relatives generated using PASC.

| The overall nucleotide sequence identity (%) | Reference Sequence | Bacteriophage | Genus within the family *Drexlerviridae* |
| --- | --- | --- | --- |
| 46.58% | NC_042122.1 | *Enterobacter* phage Ec_L1 | *Eclunavirus* |
| 42.37% | NC_031050.1 | *Esherichia* phage vB_EcoS_NBD2 | *Vilniusvirus* |
| 41.84% | MN019128.1 | *Escherichia* phage Henu7 | unclassified *Drexlerviridae* |
| 41.56% | NC_047960.1 | *Escherichia* phage vB_EcoS_IME347 | *Badaguanvirus* |
| 41.49% | MK778457.1 | *Escherichia* phage vB_EcoS_W011D | unclassified *Drexlerviridae* |
| 40.83% | NC_047825.1 | *Klebsiella* phage KOX1 | *Webervirus* |
| 40.56% | NC_042043.1 | *Escherichia* phage SRT8 | *Sertoctavirus* |
| 40.50% | NC_019509.1 | *Cronobacter* phage ESP2949-1 | *Tunavirinae* |
| 40.41% | NC_047784.1 | *Klebsiella* phage vB_KpnS_KpV522 | *Webervirus* |
| 40.08% | LT841304.1 | *Escherichia* phage vB_EcoS_swan01 | *Warwickvirus* |

**Figure S1.** Effect of temperature on the plating efficiency of phage AAS23. Each point represents the mean of three independent experiments. Error bars indicate standard deviation.

**Figure S2.** Adsorption rate of phage AAS23. Each point represents the mean of three independent experiments. Error bars indicate standard deviation.

**Figure S3.** Restriction digestion patterns of AAS23 genomic DNA. M– GeneRuler™ DNA Ladder Mix (Thermo Fisher Scientific); gDNA – undigested genomic DNA of AAS23. Methylation sensitivity: MboI (dam methylation-sensitive); EcoRII (dcm methylation-sensitive); HhaI (CpG methylation-sensitive); Bsu15I (CpG methylation-sensitive, dam methylation-sensitive); Csp6I, EcoRV, DraI, NdeI (not dam methylation-sensitive, not dcm methylation-sensitive, not CpG methylation-sensitive). On the right side of the gel is the representation of the expected restriction pattern.

**Figure S4.** Neighbor-joining tree analysis based on the alignment of the amino acid sequences of AAS23 tail fiber protein (gp24) and its closest homologues. The percentage of replicate trees in which the associated taxa clustered together in the bootstrap test is shown next to the branches.

**Figure S5.** Neighbor-joining tree analysis based on the alignment of the amino acid sequences of conserved Peptidase_S74 domain (aa 402 to 459) of AAS23 tail fiber protein (gp24) and its closest homologues. The percentage of replicate trees in which the associated taxa clustered together in the bootstrap test is shown next to the branches.
